# Organic–Inorganic Hyaluronic Acid/Sumecton Nanocomposite Hydrogels for Osteogenic Applications

**DOI:** 10.3390/gels12090851

**Published:** 2026-09-19

**Authors:** Dong Kyu Kim, Geun Jin Song, Ahyoung Yoo, Hee Sook Hwang, Min Lee, Chung-Sung Lee

**Affiliations:** 1 Department of Medical Science, Soonchunhyang University, Asan 31538, Republic of Korea; 2 Department of Pharmaceutical Engineering, Dankook University, Cheonan 31116, Republic of Korea; 3 Division of Oral and Systemic Health Sciences, School of Dentistry, University of California, Los Angeles, CA 90095, USA; 4 Department of Bioengineering, University of California, Los Angeles, CA 90095, USA; 5 Department of Pharmaceutical Engineering, Soonchunhyang University, Asan 31538, Republic of Korea; 6 Institute for Molecular Metabolism Innovation, Soonchunhyang University, Asan 31538, Republic of Korea

**Keywords:** dynamic hydrogel, catechol-functionalized hyaluronic acid, Sumecton clay nanocomposite, osteoinductive delivery, bone regenerative engineering

## Abstract

While their biocompatibility and extracellular matrix-mimetic properties make hyaluronic acid (HA)-based hydrogels attractive biomaterials for bone regenerative applications, their limited mechanical stability and insufficient osteogenic activity restrict broader utility. Here, we present an organic–inorganic nanocomposite hydrogel integrating catechol-functionalized HA with Sumecton clay (SU) nanosheets as multifunctional inorganic junction domains. Oxidative crosslinking of dopamine-functionalized HA established the primary hydrogel network, while the resulting hybrid network exhibited injectability and structural recovery following deformation. Incorporation of 2% SU increased the compressive modulus from approximately 4.6 to 6.0 kPa and delayed hydrogel degradation, while only modestly affecting equilibrium water content. The hybrid hydrogels also exhibited antimicrobial and catechol-mediated antioxidant activities, establishing a multifunctional microenvironment suited to tissue regeneration. SU-containing hydrogels promoted the osteogenic differentiation of encapsulated stem cells, as evidenced by increased alkaline phosphatase activity and matrix mineralization. SAG was incorporated into SU with a loading efficiency of approximately 95%, and SU@SAG-containing hydrogels further enhanced osteogenic differentiation and mineralization. Gene expression analysis showed increased expression of Hedgehog-associated markers together with modulation of Wnt/β-catenin-related genes, suggesting signaling responses associated with enhanced osteogenesis. Collectively, this study demonstrates an organic–inorganic network engineering strategy to develop structurally robust and biologically functional HA-based nanocomposite hydrogels for bone regenerative applications.

## 1. Introduction

In bone tissue engineering, the development of biomimetic three-dimensional (3D) matrices that can support stem cell osteogenic differentiation remains a central objective [1,2,3]. Hydrogels are particularly attractive for this purpose, because their hydrated polymeric networks can partially recapitulate the extracellular matrix-like microenvironment necessary for cell survival, proliferation, and lineage commitment [4,5,6,7]. Recent advances in hydrogel-based tissue engineering further emphasize that scaffold performance is closely linked to structure–function relationships, as three-dimensional architecture and viscoelastic properties can regulate cellular proliferation and differentiation, while hybrid network design can improve mechanical stability and degradation behavior [8]. Among various hydrogel-forming biopolymers, hyaluronic acid (HA) has been widely explored due to its excellent biocompatibility, biodegradability, hydrophilicity, and intrinsic role in tissue remodeling [9,10,11,12,13]. As a major glycosaminoglycan component of the native extracellular matrix, HA can provide a biologically permissive environment for encapsulated cells [14]. However, pristine HA-based hydrogels generally exhibit weak mechanical integrity, excessive swelling, rapid structural relaxation, and limited osteogenic bioactivity, which restrict their use as stable 3D matrices for osteogenic differentiation [5,9,15].

Chemical modification of HA offers an effective route to overcome these limitations by introducing additional molecular interactions into the hydrogel network [5]. In particular, catechol functionalization has emerged as a powerful strategy to design dynamically adaptable hydrogels inspired by mussel-derived catechol chemistry [16,17,18]. Catechol groups can participate in multiple reversible interactions, including hydrogen bonding, π–π interactions, metal coordination, and oxidative coupling [19,20,21,22]. These interactions can endow HA-based hydrogels with injectability, self-healing capability, and improved network cohesion through dynamic reversible interactions [19,20]. Reversible hydrogen bonds can reinforce hydrogel networks and dissipate mechanical stress, while their dynamic dissociation and reformation contribute to viscoelastic recovery [23]. Such dynamic characteristics are highly advantageous to construct cell-compatible hydrogels that can be handled, injected, and remodeled under physiological conditions. Nevertheless, hydrogels composed solely of organic polymer networks often remain mechanically insufficient and lack inorganic microenvironmental cues that are important for osteogenic differentiation.

The incorporation of clay nanosheets into polymeric hydrogels provides a promising strategy to bridge this gap. Layered silicate clays possess high specific surface area, nanoscale thickness, surface charge, ion–exchange capacity, and strong affinity toward hydrophilic polymers [24]. Because of these characteristics, clay nanosheets can serve not merely as passive fillers, but as active inorganic junction domains that regulate polymer chain organization, crosslinking density, hydration behavior, and stress dissipation within hydrogel networks. In addition, clay–polymer interactions can generate hybrid microenvironments that more closely resemble the organic–inorganic nature of mineralized tissues [24,25,26]. Therefore, the rational integration of clay nanosheets into polysaccharide hydrogels may provide a materials-based strategy to simultaneously improve structural stability and promote osteogenic cellular responses.

Compared with extensively investigated synthetic nanoclays such as Laponite, the application of Sumecton in biomedical hydrogel systems remains relatively underexplored [27,28,29]. In particular, its integration with catechol-functionalized HA as an organic–inorganic network and as a carrier for localized osteoinductive delivery has not been extensively investigated. Sumecton clay (SU), a synthetic smectite-type layered silicate, is particularly attractive for hydrogel engineering, due to its nanosheet morphology, high purity, swelling behavior, and capacity to form organic–inorganic hybrid structures with functional polymers [6,30]. Its negatively charged silicate layers can interact with catechol-functionalized polysaccharides through multiple noncovalent and coordination-based interactions, potentially acting as inorganic nano-crosslinking domains within a dynamic polymer network. We reasoned that such catechol–clay interactions could reinforce HA-based hydrogels, while preserving injectability and self-healing behavior. More importantly, the resulting organic–inorganic nanocomposite network may provide a favorable 3D microenvironment for stem cell osteogenic differentiation by modulating matrix stiffness, water retention, degradation behavior, and nanoscale interfacial cues.

**Figure 1:**
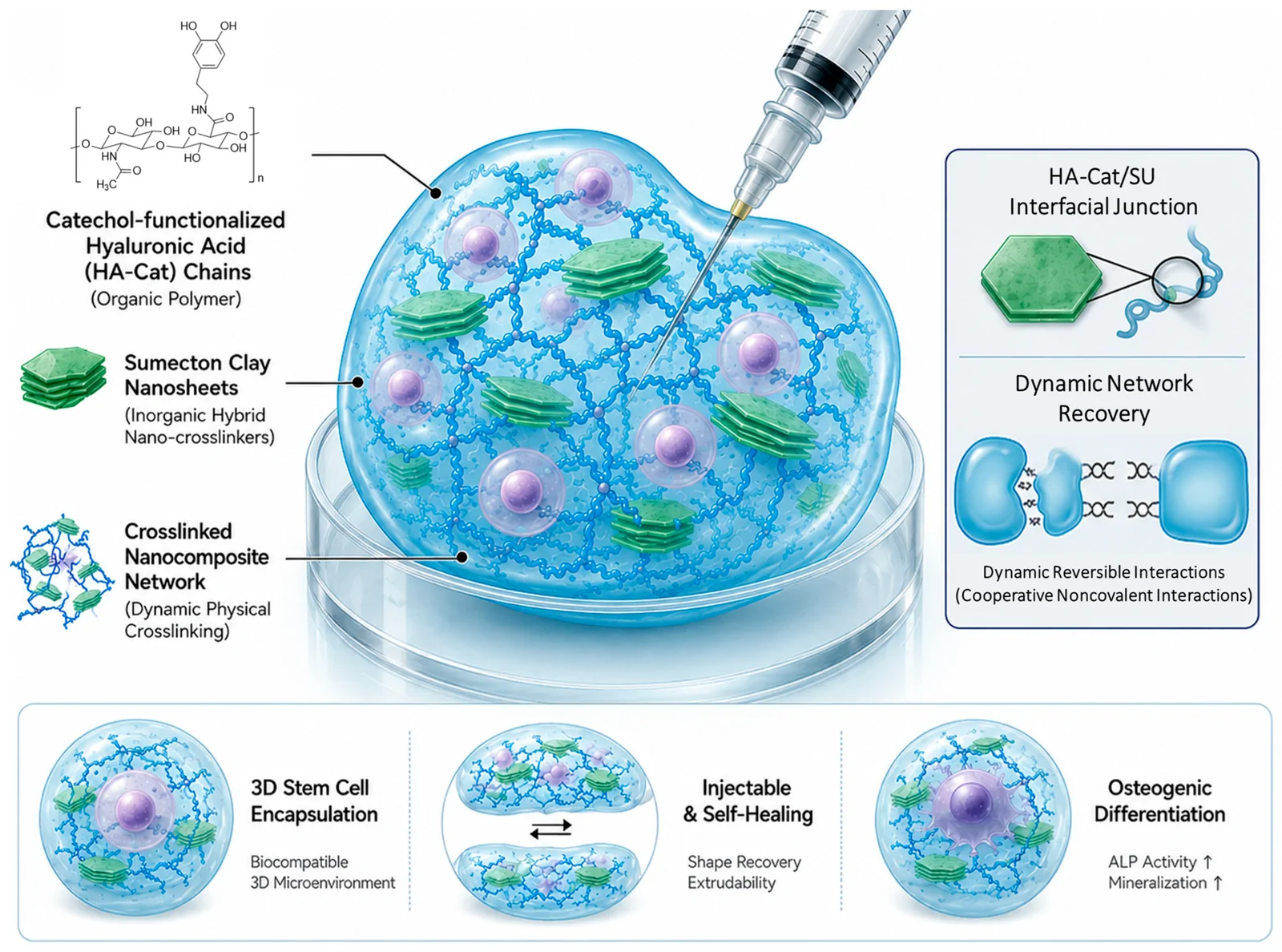
Schematic of the injectable HA-Cat/Sumecton clay (SU) nanocomposite hydrogel for osteogenic 3D stem cell culture. HA-Cat and SU nanosheets form a dynamic organic–inorganic hybrid network that exhibits injectability and self-healing behavior, while SU nanosheets reinforce the hydrogel matrix and modulate the physicochemical microenvironment for osteogenic differentiation.

Herein, we report a dynamic organic–inorganic nanocomposite hydrogel based on catechol-functionalized hyaluronic acid and SU nanosheets for osteogenic differentiation (Figure 1). In this system, catechol–modified HA forms a dynamically crosslinked organic network, while SU nanosheets function as inorganic nano-crosslinking domains that reinforce the hydrogel matrix and regulate its physicochemical properties. The resulting hydrogels were systematically evaluated in terms of structural formation, morphology, elemental composition, swelling behavior, degradation profile, mechanical performance, rheological properties, injectability, and self-healing capability. Further, 3D stem cell culture systems were used to investigate their cytocompatibility and osteogenic potential, including cell viability, alkaline phosphatase (ALP) activity, matrix mineralization, and osteogenic gene expression analyses. This study highlights SU nanosheets as multifunctional inorganic nanojunctions within catechol-functionalized polysaccharide hydrogels, enabling both physicochemical stabilization and osteogenic bioactivity for osteogenic 3D cell culture applications.

## 2. Results and Discussion

### 2.1. Structural Characterization of Catechol-Functionalized Hyaluronic Acid (HA-Cat)

**Figure 2:**
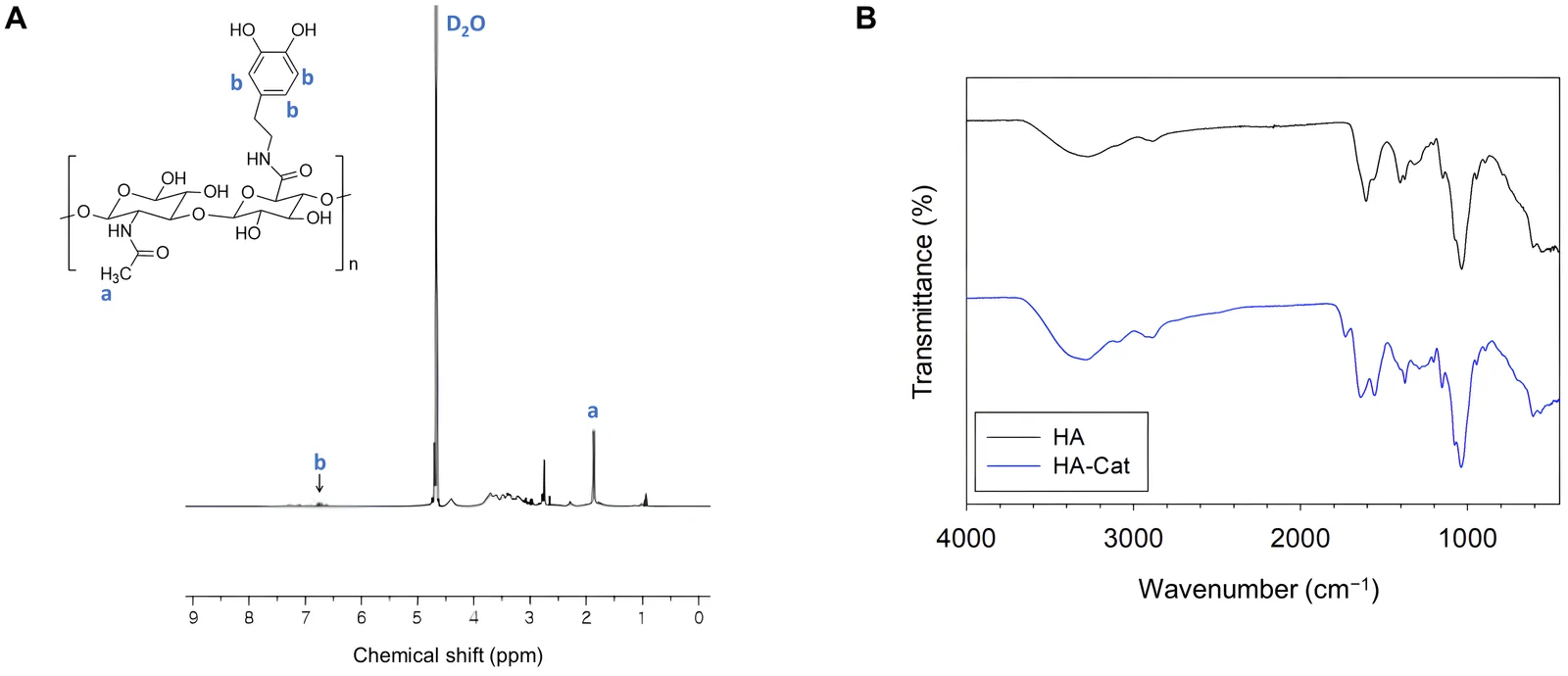
Structural characterization of catechol-functionalized hyaluronic acid (HA-Cat). (**A**) ^1^H NMR spectrum of HA-Cat in D_2_O showing characteristic catechol aromatic proton signals at 6.6–6.9 ppm. (**B**) FT-IR spectra of HA and HA-Cat showing spectral changes associated with catechol functionalization.

The ^1^H NMR spectroscopy (Figure 2A) confirmed the successful catechol functionalization of HA. Compared with the native HA, HA-Cat exhibited distinct aromatic proton signals at (6.6–6.9) ppm, corresponding to the catechol moieties of dopamine, confirming successful conjugation. FT-IR analysis further supported successful dopamine conjugation (Figure 2B). The native HA displayed characteristic broad hydroxyl stretching vibrations in the (3200–3500) cm^−1^ region, carboxylate/amide–associated bands near 1640 cm^−1^, and polysaccharide fingerprint peaks in the (1000–1150) cm^−1^ range. Following dopamine conjugation, enhanced spectral changes were observed in the amide–associated region, together with the emergence of a carbonyl-associated absorption band near 1715 cm^−1^, which may reflect changes in the carboxyl/carbonyl environment following the conjugation reaction. These spectral changes collectively support successful catechol functionalization of the HA backbone. Catechol functionalization is particularly advantageous to construct dynamic hybrid biomaterials, as catechol groups provide reversible interfacial interaction sites that can promote noncovalent molecular interactions with inorganic nanosheets. This molecular design establishes the chemical basis to construct dynamically crosslinked organic–inorganic nanocomposite hydrogels [31]. Periodate-mediated oxidation during hydrogel formation may consume a fraction of the conjugated catechol groups. Nevertheless, residual unoxidized catechol moieties may remain available to participate in reversible interactions and radical-scavenging activity, although their residual content was not independently quantified in the present study.

### 2.2. Characterization of Sumecton Clay and Osteoinductive Molecule Loading

SU was employed as a two-dimensional inorganic nanosheet serving as a dynamic inorganic nanojunction component within the catechol-functionalized hybrid hydrogel system [30]. Layered silicate clays are attractive inorganic building blocks for hybrid biomaterials due to their high specific surface area, intrinsic mechanical rigidity, and ability to accommodate bioactive guest molecules through surface adsorption and interfacial association [6]. In the present study, SU was further engineered as a carrier for smoothened agonist (SAG), an osteoinductive small molecule, to impart biofunctional activity, in addition to structural reinforcement [32,33,34].

**Figure 3:**
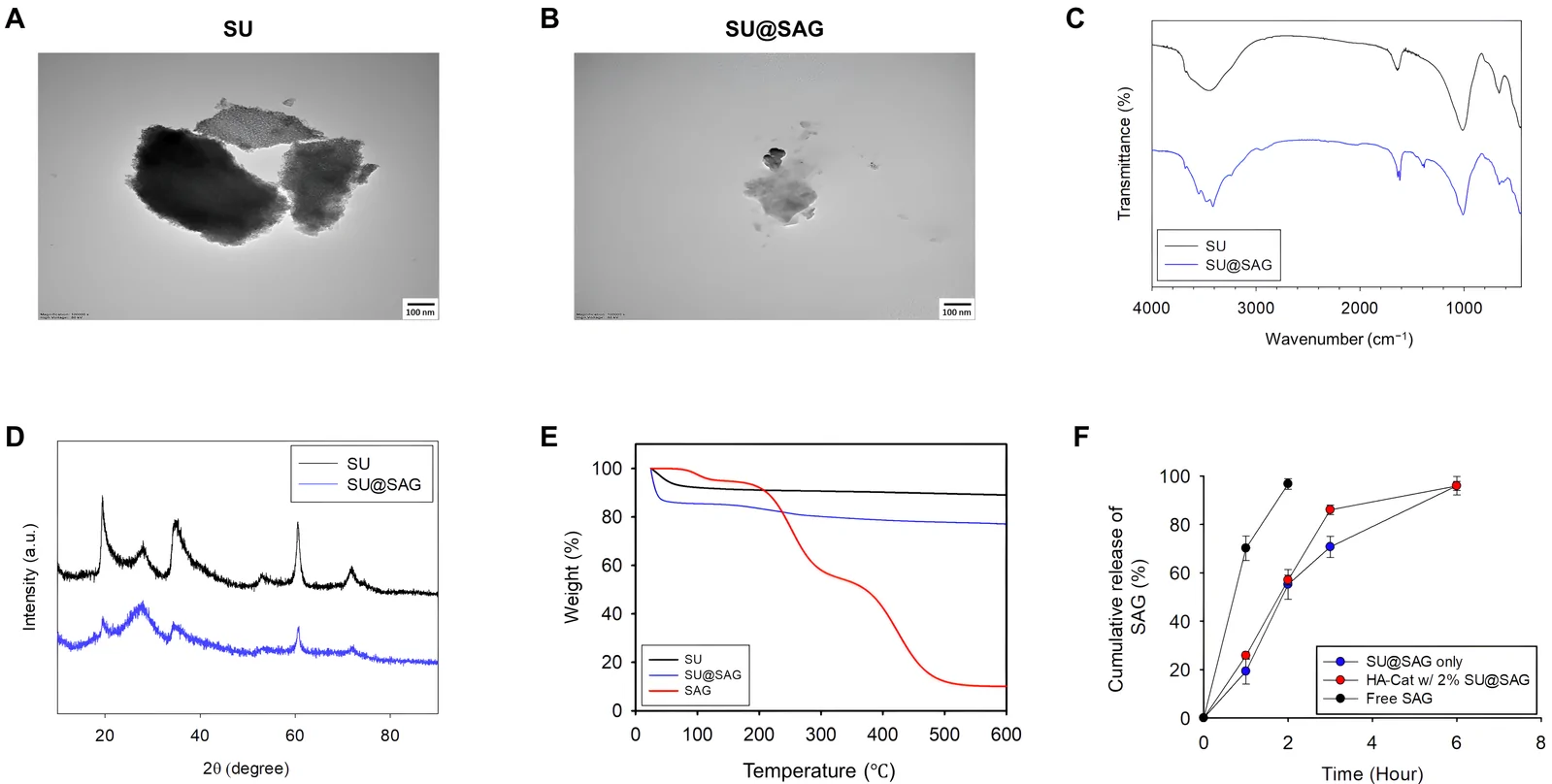
Characterization of pristine Sumecton clay (SU) and SAG-loaded Sumecton clay (SU@SAG). (**A**) TEM image of pristine SU showing the layered nanosheet morphology. Scale bar: 100 nm. (**B**) TEM image of SU@SAG showing preservation of the nanosheet morphology after SAG loading. (**C**) FT-IR spectra of SU and SU@SAG. (**D**) XRD patterns of SU and SU@SAG showing preservation of the clay framework after SAG loading. (**E**) TGA profiles of SU, SU@SAG, and free SAG. (**F**) Cumulative SAG release profiles of free SAG, SU@SAG, and HA-Cat hydrogel containing 2% SU@SAG.

TEM revealed the characteristic lamellar morphology of the pristine SU, showing densely stacked nanosheet structures consistent with layered silicate architecture (Figure 3A). Following SAG loading, SU@SAG retained the characteristic sheet-like morphology of SU (Figure 3B).

FT-IR analysis further supported interaction between SAG and the SU framework (Figure 3C). The Pristine SU exhibited characteristic hydroxyl–associated absorption bands in the (3200–3600) cm^−1^ region and strong Si–O-related vibrational features in the fingerprint region, consistent with silicate clay structures. After SAG loading, noticeable spectral perturbations were observed in both hydroxyl–associated and fingerprint regions, indicating changes in the local chemical environment of the clay framework. These spectral shifts suggest interaction between SAG molecules and SU, likely through adsorption-mediated association of SAG with the SU surface.

XRD analysis provided additional evidence of successful guest association, while confirming preservation of the clay framework (Figure 3D). The Pristine SU displayed distinct diffraction peaks characteristic of its crystalline layered silicate structure. Following SAG incorporation, these reflections were retained, but became noticeably broadened and reduced in intensity, indicating reduced structural ordering, without collapse of the layered inorganic framework. Such changes are consistent with altered interfacial interactions between SAG and the clay architecture.

TGA further corroborated successful SAG association (Figure 3E). While due to its predominantly inorganic composition, the pristine SU exhibited minimal weight loss over the examined temperature range, SU@SAG showed additional thermal decomposition behavior and increased mass loss, which was attributable to the incorporated organic SAG molecules. The intermediate degradation profile of SU@SAG, positioned between the pristine SU and free SAG, further supports the successful association of SAG with the clay nanosheets.

The SAG loading efficiency of SU@SAG was 95.3 ± 4.2%, as determined by HPLC-UV analysis. Both SU@SAG and the HA–Cat hydrogel containing 2% SU@SAG showed progressive SAG release, reaching approximately 95% cumulative release within 6 h (Figure 3F). These results confirmed that SAG remained releasable following incorporation into the hydrogel system. For comparison, free SAG reached nearly complete release within 2 h under the same experimental conditions, whereas SU-associated SAG showed a comparatively delayed release profile, supporting the role of SU as a reservoir for modulating SAG release.

Collectively, these results confirm that SU serves as a multifunctional inorganic building block that can simultaneously reinforce the hybrid hydrogel network while accommodating osteoinductive guest molecules. This dual structural and biofunctional role provides a rational foundation to construct dynamic organic–inorganic nanocomposite hydrogels for osteogenic applications.

### 2.3. Injectable, Moldable, and Self-Healing Behavior of the HA-Cat/SU Nanocomposite Hydrogels

**Figure 4:**
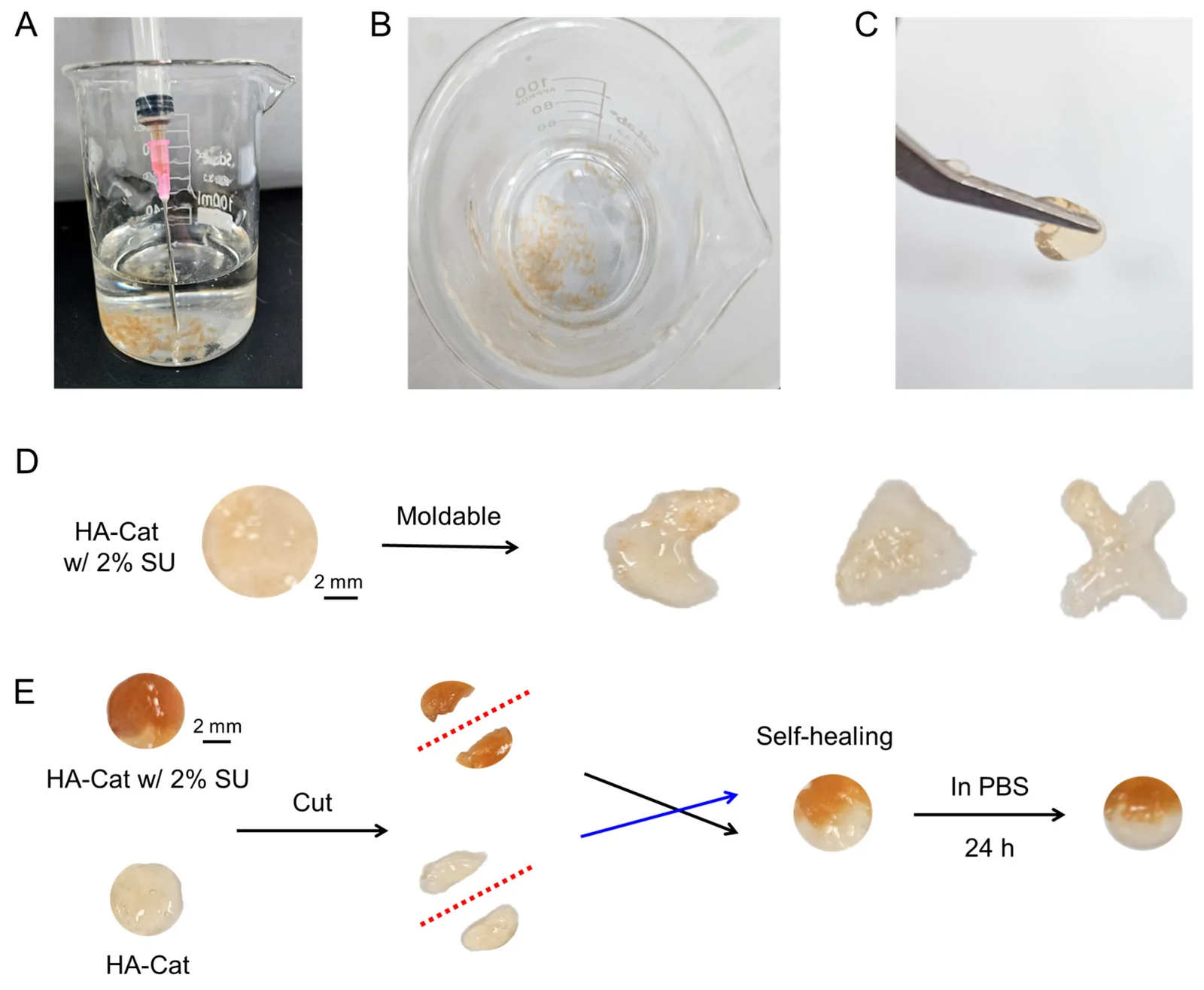
Macroscopic injectability, moldability, and self-healing behavior of the HA-Cat/SU nanocomposite hydrogels. (**A**) Hydrogel injection through a syringe needle into 0.01 M PBS (pH 7.4). (**B**) Cohesive hydrogel formation after extrusion. (**C**) Mechanical integrity of the hydrogel. (**D**) Shape adaptability after injection and molding into various geometries. (**E**) Self-healing behavior of HA-Cat and HA-Cat/SU hydrogels evaluated by cut-and-rejoin experiments. Scale bars: 2 mm.

The practical applicability of injectable hydrogels depends on their ability to undergo deformation during administration while recovering structural integrity after placement [19,35]. The macroscopic handling behavior and dynamic rheological properties of the HA-Cat/SU nanocomposite hydrogels were therefore evaluated. The HA-Cat/SU hydrogel could be smoothly extruded through a syringe needle into PBS, where it maintained a cohesive gel-like structure, without immediate dispersion or fragmentation (Figure 4A,B). This behavior indicates that under shear stress, the hydrogel network can undergo transient deformation, and after extrusion, subsequently recover. The hydrogel also exhibited sufficient macroscopic cohesion to be lifted with tweezers (Figure 4C), suggesting that the incorporation of SU nanosheets contributes to the structural stability of the HA-Cat network. In addition, the injected hydrogel could be molded into various shapes, demonstrating shape adaptability and handling flexibility (Figure 4D). Self-healing behavior was further examined using cut-and-rejoin experiments (Figure 4E). After being divided into two pieces, the hydrogel fragments were brought into contact and reassembled into an integrated construct, without additional external triggers. This recovery likely arises from the combined contribution of reversible interactions within the HA-Cat/SU network, including hydrogen bonding, catechol-associated interactions, and physical polymer–clay interactions. These interactions likely act cooperatively, and their individual contributions were not distinguished in the present study.

**Figure 5:**
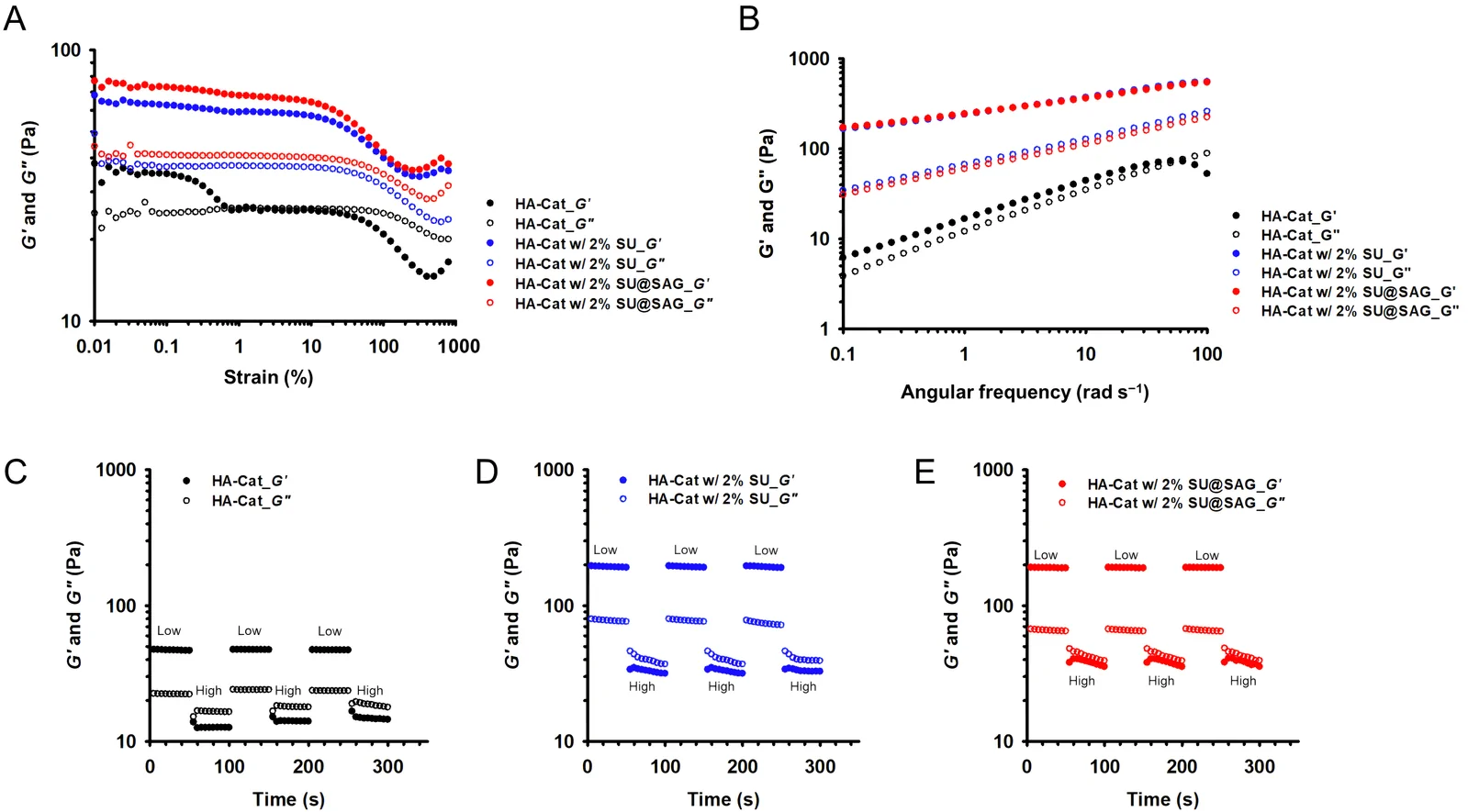
Rheological characterization of the HA-Cat/SU nanocomposite hydrogels. (**A**) Strain sweep analysis of HA-Cat, HA-Cat with 2% SU, and HA-Cat with 2% SU@SAG hydrogels. (**B**) Frequency sweep analysis of the hydrogel formulations. (**C**–**E**) Cyclic high–low strain tests showing structural recovery of HA-Cat, HA-Cat with 2% SU, and HA-Cat with 2% SU@SAG hydrogels.

Rheological analysis was performed to further investigate the viscoelastic properties of the hydrogels (Figure 5). In the strain sweep test, all hydrogel formulations showed storage modulus (*G*′) values higher than the loss modulus (*G*″) within the linear viscoelastic region, confirming the formation of elastic gel networks (Figure 5A). Compared to HA-Cat alone, HA-Cat containing 2% SU exhibited increased *G*′ values, indicating that SU nanosheets function as inorganic nano-crosslinking domains that enhance network elasticity. HA-Cat containing 2% SU@SAG showed the highest *G*′ among the tested groups, suggesting that SAG loading did not interfere with the reinforcing function of SU, and might further contribute to polymer–clay interfacial interactions.

Frequency sweep analysis also showed that *G*′ remained higher than *G*″ over the tested angular frequency range (Figure 5B), confirming stable viscoelastic gel behavior. The relatively weak frequency dependence of *G*′ suggests that the hydrogels possess physically associated networks that were maintained by dynamic, reversible junctions, rather than purely permanent crosslinks. The increased moduli in SU- and SU@SAG-containing hydrogels further support the role of clay nanosheets as reinforcing junction points within the HA-Cat matrix.

To evaluate self-recovery under repeated deformation, cyclic strain tests were conducted (Figure 5C–E). Under high strain, the hydrogel networks were temporarily disrupted, as reflected by a marked decrease in *G*′. Upon returning to low strain, *G*′ rapidly recovered, and remained higher than *G*″ over repeated cycles. This reversible sol–gel transition-like behavior demonstrates that the hydrogels can repeatedly undergo mechanical disruption and structural recovery [9]. Importantly, the recovery behavior was retained after the incorporation of SU and SU@SAG, indicating that clay-mediated reinforcement does not compromise the dynamic self-healing nature of the HA-Cat network.

Collectively, these results demonstrate that the HA-Cat/SU nanocomposite hydrogel achieves a balanced combination of injectability, moldability, mechanical cohesion, and self-healing viscoelastic behavior. The SU nanosheets serve as inorganic reinforcing domains within the catechol-functionalized HA network, enhancing elastic stability, while maintaining the reversible interactions necessary for dynamic network adaptation.

### 2.4. SU-Mediated Physicochemical Stabilization and the Structural Reinforcement of Hybrid Hydrogels

**Figure 6:**
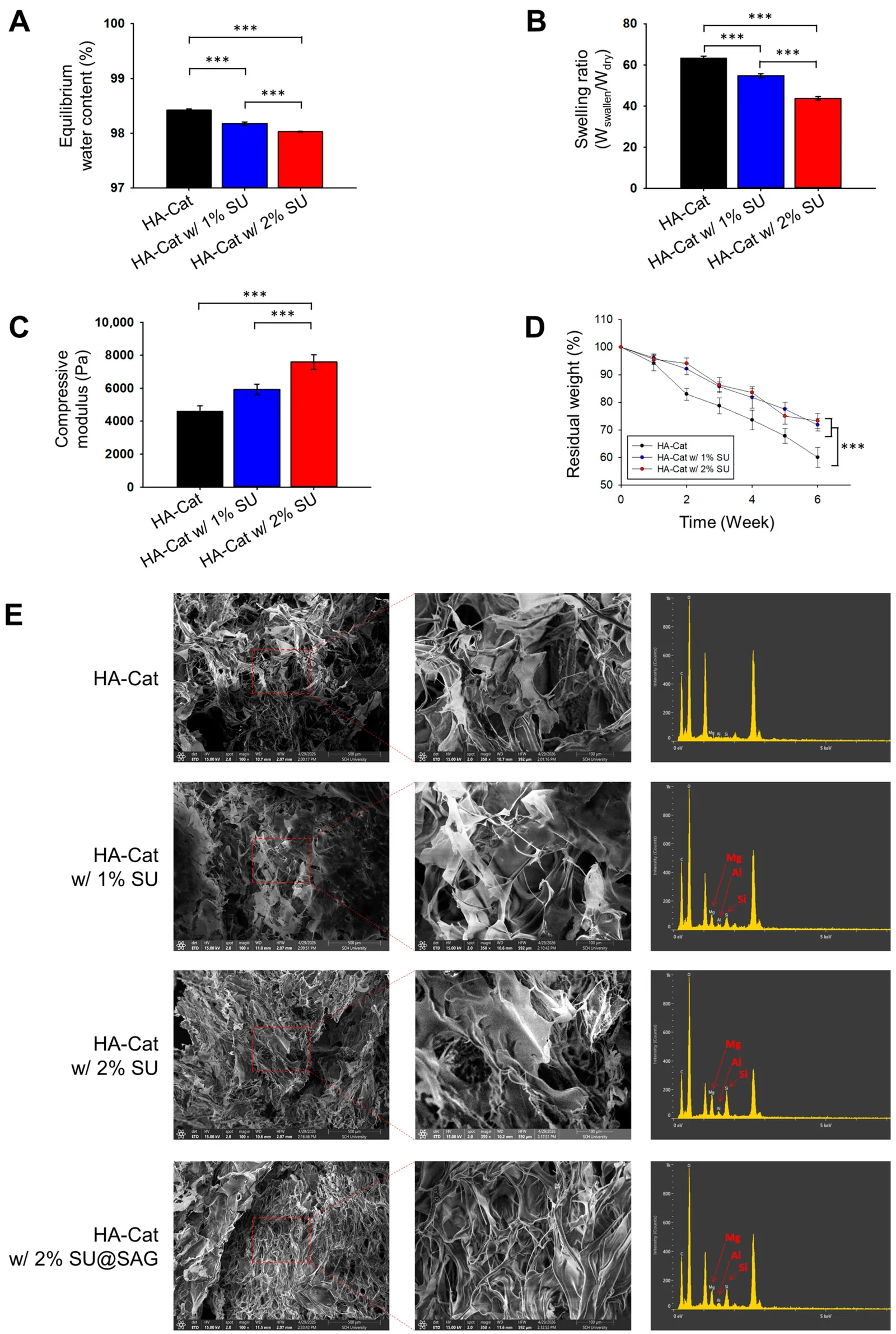
Physicochemical and microstructural characterization of the HA-Cat/SU hybrid hydrogels. (**A**) Equilibrium water content. (**B**) Equilibrium swelling ratio. (**C**) Compressive modulus. (**D**) In vitro degradation profiles over 6 weeks. (**E**) Representative FE-SEM images of freeze-dried hydrogels with corresponding EDS spectra showing SU-derived elements (Mg, Al, and Si). Scale bars as indicated. Data are presented as mean ± SD. Panels (**A**–**C**) were analyzed using one-way ANOVA followed by Tukey’s post hoc test, and panel (**D**) using two-way ANOVA with formulation and time as independent factors (*** *p* < 0.001).

To further investigate the influence of SU incorporation on the structural stability of the HA-Cat hydrogel network, the physicochemical properties of the hybrid hydrogels were systematically evaluated, including their hydration behavior, swelling, compressive mechanical properties, degradation kinetics, and internal microstructure. All hydrogel formulations maintained high equilibrium water content, consistent with the highly hydrophilic nature of the HA-based polymer network (Figure 6A). However, incorporation of SU resulted in a modest yet statistically significant reduction in water content, compared with HA-Cat alone. This decrease suggests that incorporation of the inorganic SU phase partially limits water uptake by promoting a more constrained network architecture through additional physical interactions within the polymer matrix.

A more pronounced effect was observed in the swelling behavior (Figure 6B). The HA-Cat hydrogel exhibited the highest swelling ratio, whereas incorporation of (1 and 2) % SU significantly reduced hydration-driven expansion. This reduction indicates that during hydration, SU incorporation restricts polymer chain relaxation, resulting in improved dimensional stability. Such behavior is advantageous for injectable biomaterials, where excessive swelling after placement may compromise geometric fidelity and local structural stability [36,37]. Mechanistically, physical associations between HA-Cat chains and SU nanosheet surfaces may act as additional physical crosslinking junctions, restricting polymer-chain mobility and network expansion during hydration. This increased network constraint can account for the reduced swelling, enhanced compressive resistance, and delayed hydration-mediated degradation observed after SU incorporation.

The reinforcing effect of SU was further confirmed by compressive mechanical testing (Figure 6C). The compressive modulus increased significantly in a concentration-dependent manner following SU incorporation, demonstrating clear mechanical strengthening of the hydrogel network. This trend is consistent with the rheological data presented earlier, and suggests that the SU phase functions as an effective inorganic reinforcing domain within the catechol-functionalized polymer matrix. The absolute compressive modulus values were higher than the oscillatory shear moduli because these measurements probe the hydrogel response under distinct deformation modes and testing conditions; therefore, the two modulus values were not directly compared quantitatively. The increased stiffness likely arises from restricted polymer mobility and enhanced structural resistance to compressive deformation imparted by the incorporated inorganic phase [36,38].

The effect of SU incorporation on long-term structural integrity was further examined through in vitro degradation analysis (Figure 6D). HA-Cat alone exhibited progressive mass loss over the 6 weeks incubation period, whereas SU-containing hydrogels retained significantly greater residual mass throughout the study. The slower degradation behavior suggests that the denser hybrid network formed in the presence of SU provides increased resistance against hydration-mediated structural disintegration. Notably, the 2% SU formulation showed the highest residual mass, indicating a concentration–dependent stabilization effect. As the present degradation study was conducted under hydrolytic conditions in PBS, future studies should further evaluate hydrogel degradation under physiologically relevant enzymatic conditions, including hyaluronidase.

Microstructural and compositional analyses provided additional insight into the structural effects of SU incorporation within the HA-Cat hydrogel network. FE-SEM analysis revealed clear microstructural changes following SU incorporation (Figure 6E). However, because the hydrogels were freeze-dried prior to FE-SEM analysis, the observed pore morphology may be influenced by ice-crystal templating during sample preparation and should not be considered a direct representation of the hydrated hydrogel network. HA-Cat alone displayed a relatively loose and porous fibrillar architecture with heterogeneous internal voids, whereas SU-containing hydrogels exhibited denser and more compact internal morphologies with more condensed interconnected matrix structures. These structural changes are consistent with the suppressed swelling behavior, delayed degradation, and enhanced compressive stiffness observed in Figure 6A–D, suggesting that SU contributes to increased network compactness and structural stabilization.

Representative EDS spectra further supported the presence of inorganic clay–derived components within the hybrid hydrogels (Figure 6E), while quantitative elemental analysis (Table 1) focused on the characteristic SU-derived inorganic elements to provide additional compositional confirmation. HA-Cat consisted predominantly of carbon and oxygen with negligible inorganic elemental content, whereas SU-containing formulations exhibited clear detection of characteristic Mg, Al, and Si elements originating from the SU structure. Increasing SU content was associated with higher detectable levels of inorganic elemental signals, particularly Si and Al, supporting greater integration of the inorganic phase within the polymer matrix. The HA-Cat with 2% SU@SAG formulation similarly retained detectable Mg, Al, and Si signals, consistent with retention of the inorganic SU phase following SAG loading.

Collectively, these findings demonstrate that SU incorporation reinforces the HA-Cat hydrogel network at both the structural and compositional levels, resulting in improved physicochemical stability and mechanical robustness suitable for subsequent biological evaluation.

### 2.5. Cytocompatibility and Osteogenic Responses of the HA-Cat/SU Nanocomposite Hydrogels in 3D Culture

**Figure 7:**
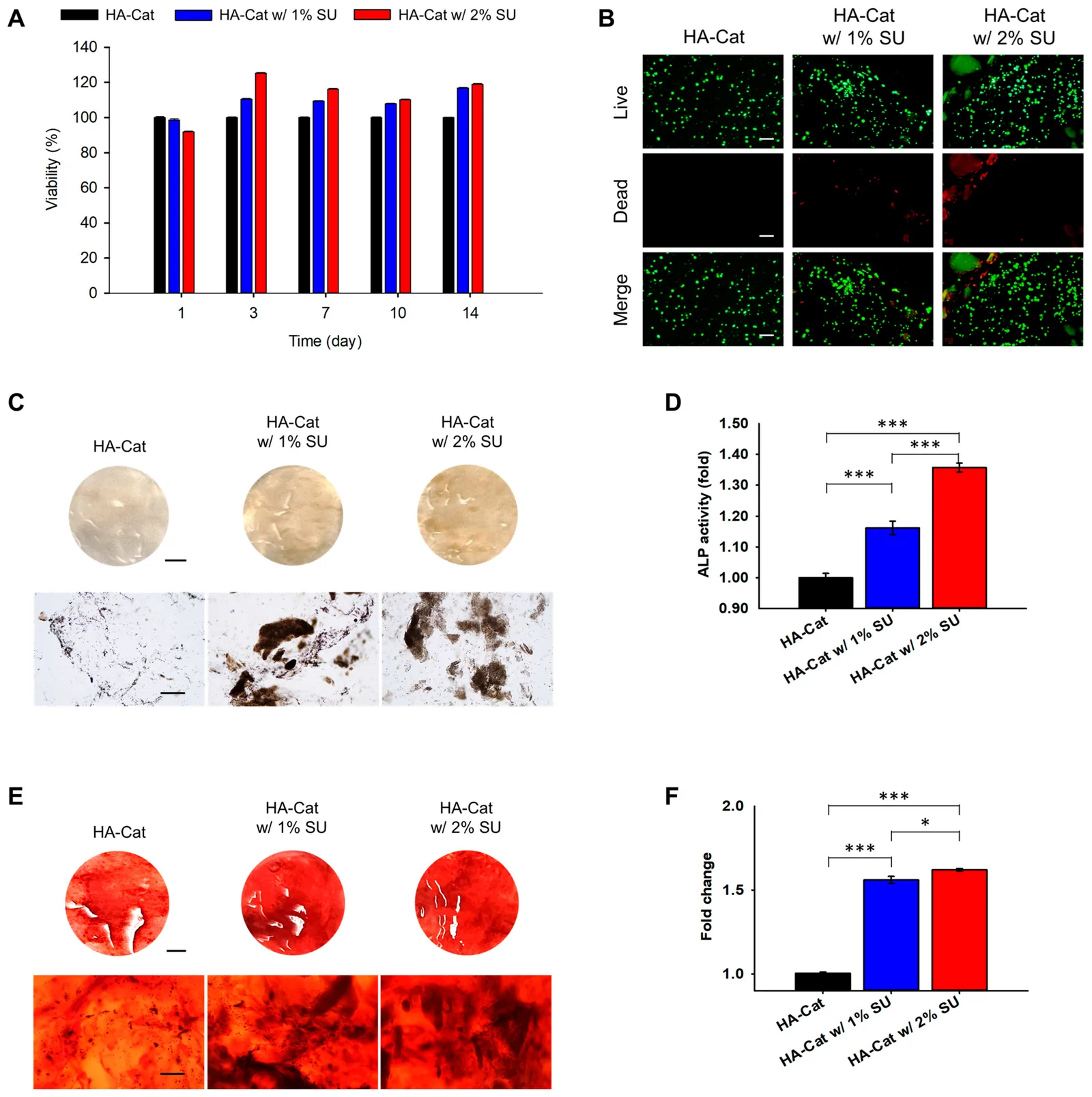
Cytocompatibility and osteogenic effects of SU-containing HA-Cat hybrid hydrogels in 3D BMSC culture. (**A**) Cell viability over 14 d. (**B**) Live/dead staining of encapsulated BMSCs (live, green; dead, red). (**C**) Representative ALP staining images. (**D**) Quantitative ALP activity. (**E**) Representative Alizarin Red S staining images. (**F**) Quantification of ARS staining. Data are presented as mean ± SD (*n* = 3). Scale bars in C and E indicate 1 mm (top) and 100 μm (bottom). Statistical significance was analyzed using one-way ANOVA with Tukey’s post hoc test (* *p* < 0.05, *** *p* < 0.001).

Following confirmation that SU incorporation improved the physicochemical stability and structural integrity of HA-Cat hydrogels, we next investigated whether these material-level modifications could beneficially influence biological responses relevant to bone regeneration using a 3D cell-laden hydrogel culture model. Cytocompatibility was first evaluated using mouse bone marrow stromal cells (BMSCs) encapsulated within HA-Cat hydrogels containing varying concentrations of SU (Figure 7A). All hydrogel formulations maintained excellent cell viability throughout the 14 days culture period, with no evidence of cytotoxicity. Although residual sodium periodate may cause cytotoxicity, the hydrogels were thoroughly washed with PBS before biological evaluation to remove residual oxidant and soluble reaction products. SU-containing hydrogels exhibited comparable or slightly higher normalized cell viability relative to HA-Cat, indicating that incorporation of the inorganic SU phase did not compromise cytocompatibility within the 3D hydrogel environment.

Live/dead fluorescence imaging further supported these findings (Figure 7B). Across all groups, most encapsulated cells exhibited strong green fluorescence with minimal red fluorescence, confirming high cell survival throughout the hydrogel matrix. Notably, SU-containing hydrogels displayed slightly greater cellular density and more homogeneous cellular distribution compared to HA-Cat alone, suggesting that the modified physicochemical microenvironment created by SU incorporation supports improved cell maintenance within the 3D matrix.

To determine whether SU contributes to osteogenic differentiation, early and late osteogenesis markers were subsequently evaluated under osteogenic induction conditions. ALP staining and quantitative activity analysis demonstrated a clear concentration–dependent enhancement in early osteogenic differentiation following SU incorporation (Figure 7C,D). Compared with HA-Cat alone, both (1 and 2) % SU-containing hydrogels significantly increased ALP activity, with the highest response observed in the 2% SU group. These findings suggest that beyond simple structural reinforcement, SU contributes intrinsic osteoconductive functionality.

Consistent with the early differentiation results, ARS staining revealed enhanced mineralized matrix deposition in SU-containing hydrogels following osteogenic induction (Figure 7E,F). The 2% SU formulation showed the highest mineralization level, indicating that incorporation of the inorganic SU phase supports progression toward mature osteogenic mineralization within the 3D hydrogel environment. These effects may arise from multiple contributing factors, including improved structural stability, reduced hydrogel over-swelling, and altered cell–matrix interactions associated with the incorporated inorganic mineral phase. These results establish SU as a multifunctional inorganic module that can simultaneously improve hydrogel physicochemical performance, and provide intrinsic osteoconductive support for 3D bone regenerative cell culture.

### 2.6. Enhanced Osteogenic Performance of the SAG-Loaded HA-Cat/SU Nanocomposite Hydrogels in 3D Culture

**Figure 8:**
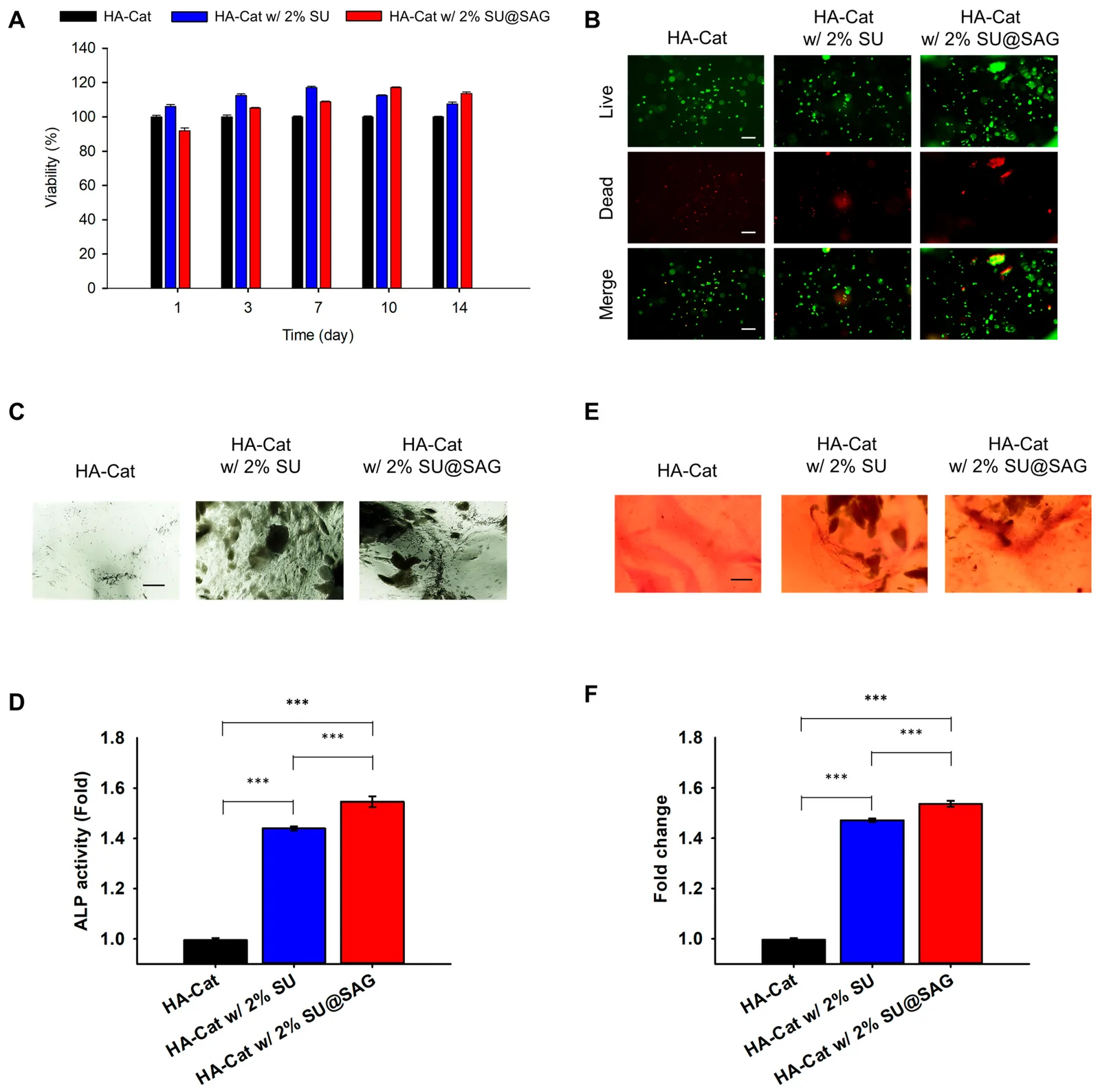
Osteogenic performance of SAG-loaded HA-Cat/SU nanocomposite hydrogels in 3D BMSC culture. (**A**) Cell viability over 14 d. (**B**) Live/dead staining of encapsulated BMSCs (live, green; dead, red). (**C**) Representative ALP staining images. (**D**) Quantitative ALP activity. (**E**) Representative Alizarin Red S staining images. (**F**) Quantitative analysis of mineralization. Data are presented as mean ± SD (*n* = 3). Scale bars are 100 µm. Statistical significance was analyzed using one-way ANOVA with Tukey’s post hoc test (*** *p* < 0.001).

Having established the intrinsic osteoconductive contribution of SU in the 3D BMSC-laden hydrogel system, we next investigated whether incorporation of the osteoinductive small molecule SAG within the inorganic clay phase could further enhance the biological functionality of the hybrid nanocomposite hydrogels. Cytocompatibility of the SAG-loaded formulation was first evaluated using BMSCs encapsulated within the hydrogel matrix under 3D culture conditions (Figure 8A). All hydrogel formulations maintained excellent cellular viability throughout the 14 d culture period, with no evidence of measurable cytotoxicity. The HA-Cat w/2% SU and HA-Cat w/2% SU@SAG groups exhibited comparable or slightly higher normalized cell viability relative to HA-Cat, indicating that the incorporation of SU and subsequent SAG loading did not compromise the cytocompatible nature of the hydrogel system.

Live/dead fluorescence imaging further confirmed robust cellular survival throughout the encapsulated 3D constructs (Figure 8B). Predominantly green fluorescence with minimal red staining was observed across all groups, indicating the high viability of encapsulated BMSCs. Notably, the HA-Cat w/2% SU@SAG group exhibited a visibly denser viable cell population, suggesting that incorporation of SAG within the hydrogel matrix supports favorable cellular responses.

Following SAG incorporation, functional osteogenic differentiation assays under osteogenic induction conditions demonstrated a clear enhancement. ALP staining and quantitative activity analysis revealed significantly elevated early osteogenic differentiation in the HA-Cat w/2% SU@SAG group, compared to both HA-Cat alone, and the SU-only group (Figure 8C,D). While SU itself promoted osteogenic differentiation through its inorganic osteoconductive microenvironment, incorporation of SAG provided an additional stimulatory effect, indicating the successful preservation of osteoinductive bioactivity following loading within the clay reservoir.

Consistent with the early differentiation findings, mineralization assays demonstrated a similar enhancement pattern (Figure 8E,F). ARS staining revealed markedly increased calcium deposition in the HA-Cat w/2% SU@SAG group, indicating the enhanced late-stage osteogenic maturation of encapsulated BMSCs. Importantly, this enhancement exceeded that achieved by the SU-only formulation, suggesting that the hybrid platform combines osteoconductive support from the inorganic scaffold with osteogenic effects associated with SAG incorporation.

**Figure 9:**
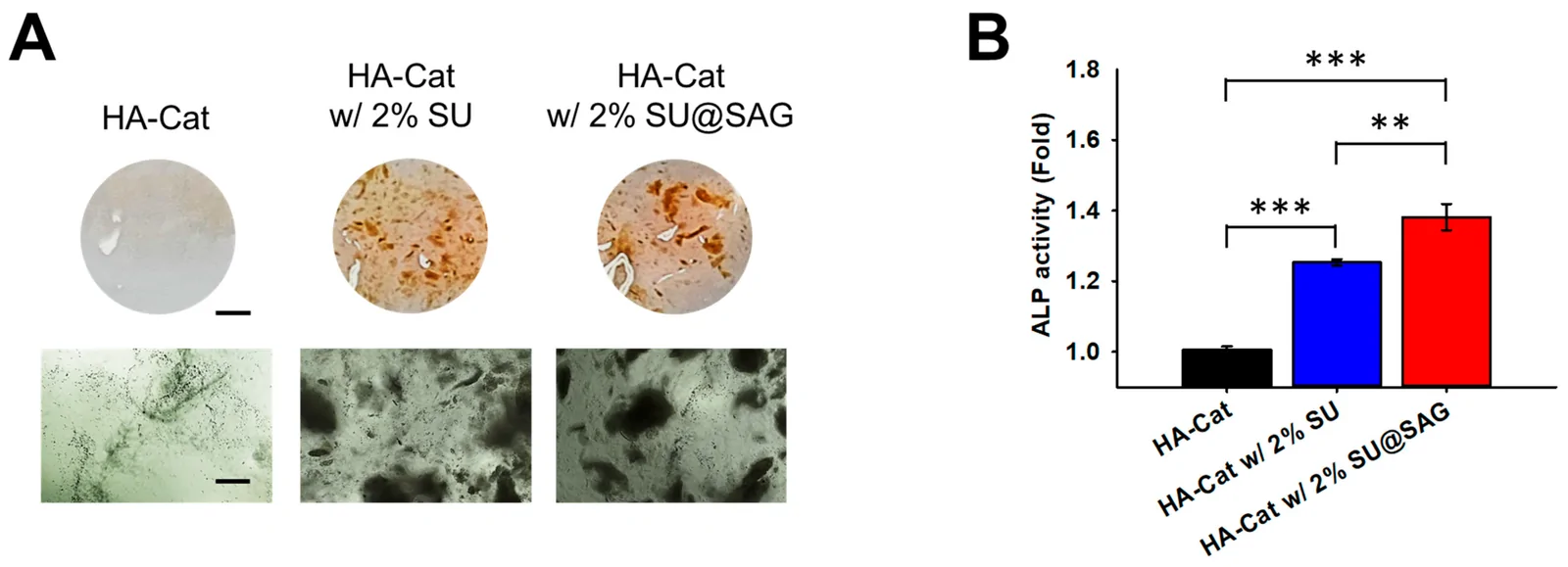
Osteogenic differentiation under growth medium conditions without exogenous osteogenic supplements. (**A**) Representative ALP staining images of BMSCs cultured in HA-Cat, HA-Cat w/2% SU, and HA-Cat w/2% SU@SAG hydrogels. (**B**) Quantitative ALP activity. Data are presented as mean ± SD (*n* = 3). Scale bars are 100 µm. Statistical significance was analyzed using one-way ANOVA with Tukey’s post hoc test (** *p* < 0.01, *** *p* < 0.001).

To further determine whether the observed enhancement reflected intrinsic material-driven bioactivity, rather than solely responsiveness to exogenous osteogenic supplements, early osteogenic differentiation was also evaluated under standard growth medium conditions lacking osteogenic induction factors (Figure 9). Under these non-inductive conditions, HA-Cat alone exhibited minimal baseline ALP activity, whereas SU incorporation significantly increased ALP expression, consistent with its intrinsic osteoconductive effect. Notably, the HA-Cat w/2% SU@SAG group demonstrated a further significant increase in ALP activity relative to the SU-only group, even in the absence of osteogenic supplements. These findings indicate that SAG retained its biological functionality following incorporation within the hybrid matrix, and actively promoted osteogenic commitment, independent of external induction cues.

Collectively, these results demonstrate that the HA-Cat/SU@SAG nanocomposite hydrogel functions not merely as a structurally reinforced scaffold, but as a biologically instructive therapeutic matrix that can simultaneously provide physicochemical stabilization, osteoconductive microenvironmental support, and SAG-associated osteoinductive activity for bone regenerative applications.

### 2.7. Osteogenic Gene Expression

**Figure 10:**
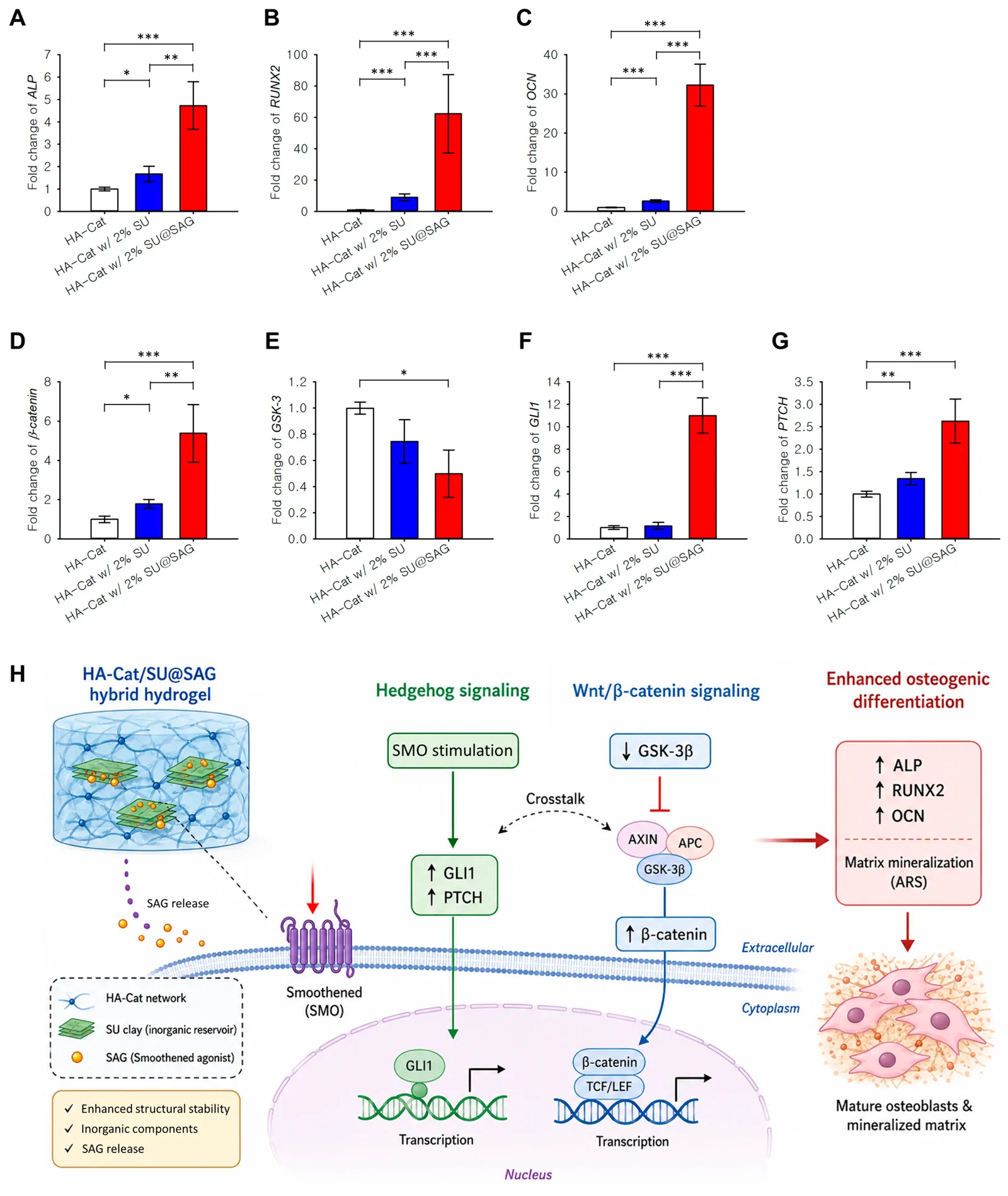
Osteogenesis- and signaling-related gene expression in BMSCs cultured within SAG-loaded HA-Cat/SU nanocomposite hydrogels. Quantitative expression of (**A**) *ALP*, (**B**) *RUNX2*, (**C**) *OCN*, (**D**) *β-catenin*, (**E**) *GSK-3β*, (**F**) *GLI1*, and (**G**) *PTCH*. Data are presented as mean ± SD (*n* = 3). Statistical significance was analyzed using one-way ANOVA with Tukey’s post hoc test (* p < 0.05, ** p < 0.01, *** *p* < 0.001). (**H**) Proposed mechanism associated with the osteogenic response of the HA-Cat/SU@SAG nanocomposite hydrogel.

To further elucidate the molecular mechanisms underlying the enhanced osteogenic performance of the SAG-loaded hybrid hydrogel, expression levels of key osteogenic differentiation markers and signaling pathway mediators were analyzed in BMSCs encapsulated within the hydrogel matrix (Figure 10). Consistent with the phenotypic osteogenic differentiation results, the HA-Cat w/2% SU@SAG group exhibited significantly elevated expression of canonical osteogenic markers, including ALP, RUNX2, and OCN, compared to both HA-Cat and SU-only groups (Figure 10A–C). The marked upregulation of RUNX2 and OCN suggests enhanced osteogenic commitment and progression toward mature osteoblastic differentiation.

Because SAG is a well-established Smoothened agonist that can activate Hedgehog signaling, downstream pathway-associated gene expression was examined to investigate the mechanistic basis of this enhanced osteogenic response [32,39]. Expression of GLI1 and PTCH was significantly upregulated in the SU@SAG group (Figure 10F,G), consistent with modulation of Hedgehog-related gene expression following SAG delivery. These findings indicate that the incorporated SAG retained biological functionality following loading within the SU-based inorganic reservoir. The SU@SAG formulation also significantly increased *β-catenin* expression and reduced *GSK-3β* expression relative to HA-Cat (Figure 10D,E), suggesting modulation of Wnt/β-catenin-related signaling [4,40]. Given the well-established crosstalk between Hedgehog and Wnt signaling during osteogenesis, these gene-expression patterns suggest that SAG delivery may promote osteogenic differentiation through coordinated regulation of Hedgehog- and Wnt/β-catenin-related signaling.

Taken together, these gene-expression findings align with the observed enhancement in ALP activity and matrix mineralization, suggesting that the HA-Cat/SU@SAG nanocomposite hydrogel promotes osteogenesis through both structural osteoconductive support and modulation of osteogenesis-related signaling. Figure 10H presents a proposed mechanistic schematic summarizing these interactions. Specifically, SAG release from the SU-based inorganic reservoir may stimulate Smoothened and enhance Hedgehog-related signaling, as reflected by the upregulation of GLI1 and PTCH, while the observed reduction in *GSK-3β* and increase in β-catenin expression suggest concurrent modulation of Wnt/β-catenin-related signaling. These coordinated gene-expression patterns provide a plausible mechanistic basis for the enhanced osteogenic differentiation and mineralization observed in the hybrid hydrogel system. Further in vivo studies will be required to validate the therapeutic potential of the developed hydrogel system.

### 2.8. Antimicrobial and Antioxidant Functionalities of Hybrid Hydrogels

**Figure 11:**
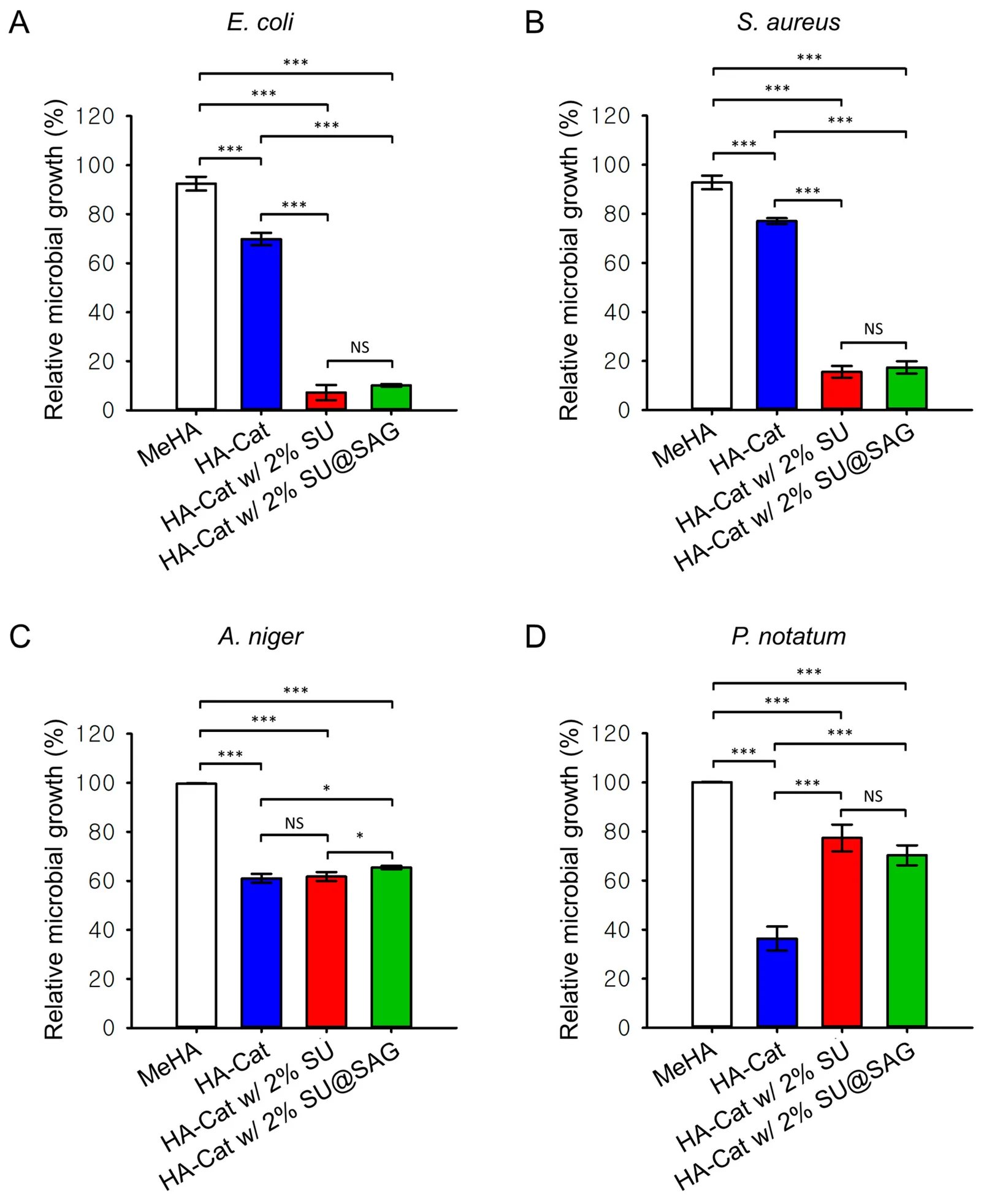
Antimicrobial activity of HA-Cat/SU nanocomposite hydrogels. Relative microbial growth of (**A**) *Escherichia coli*, (**B**) *Staphylococcus aureus*, (**C**) *Aspergillus niger*, and (**D**) *Penicillium notatum* following incubation with MeHA, HA-Cat, HA-Cat w/2% SU, and HA-Cat w/2% SU@SAG hydrogels. MeHA was used as the catechol- and SU-free control. Data are presented as mean ± SD (*n* = 3). Statistical significance was analyzed using one-way ANOVA with Tukey’s post hoc test (* *p* < 0.05, *** *p* < 0.001; NS, not significant).

The multifunctional biological performance of the hybrid hydrogel system was further evaluated in terms of antimicrobial protection and antioxidative activity, both of which are important considerations in bone regenerative biomaterial design. The antimicrobial activity of the hydrogels was assessed against representative bacterial and fungal species, including *Escherichia coli* (*E. coli*), *Staphylococcus aureus* (*S. aureus*), *Aspergillus niger* (*A. niger*), and *Penicillium notatum* (*P. notatum*) (Figure 11). The MeHA control, lacking both catechol functionality and SU incorporation, exhibited minimal antimicrobial activity, while HA-Cat showed moderate inhibitory effects compared to MeHA, potentially associated with catechol-mediated interfacial interactions [9]. SU incorporation enhanced growth inhibition against the tested bacterial species, *E. coli* and *S. aureus*. In contrast, SU incorporation did not enhance antifungal activity against *A. niger* and *P. notatum*, with HA-Cat showing comparable or greater inhibitory effects. These results indicate that the antimicrobial contribution of SU was more pronounced against the tested bacterial species rather than representing a uniform broad-spectrum effect. No significant difference was observed between the SU-only and SU@SAG groups for the bacterial species, indicating that SAG incorporation did not substantially alter the SU-associated bacterial growth-inhibitory effect. Overall, these findings suggest that the antimicrobial behavior of the hybrid system is species-dependent, with SU-associated enhancement primarily observed against the tested bacterial species.

**Figure 12:**
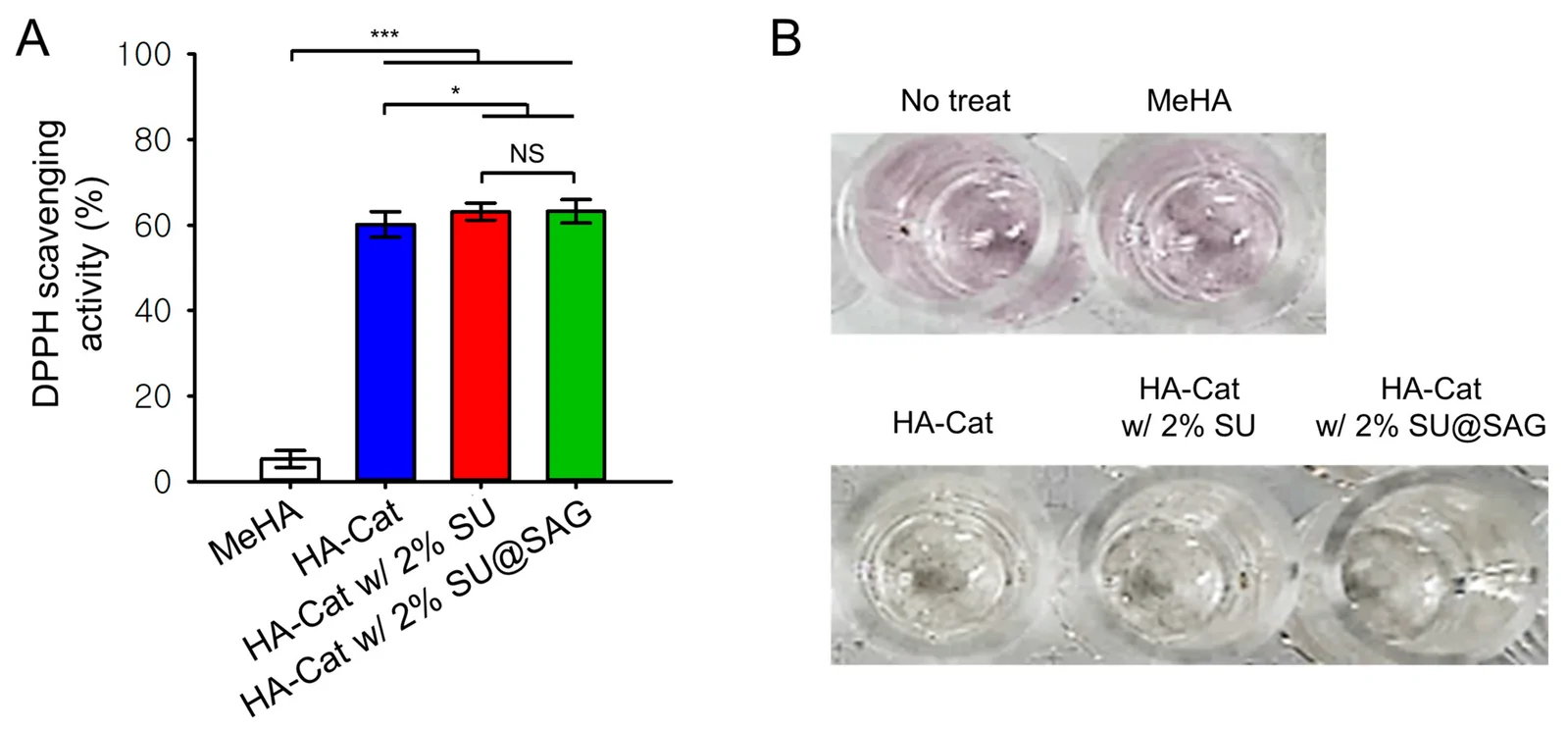
Antioxidant activity of hybrid hydrogels evaluated by the DPPH radical scavenging assay. (**A**) DPPH radical scavenging activity of MeHA, HA-Cat, HA-Cat w/2% SU, and HA-Cat w/2% SU@SAG hydrogels. MeHA was used as the catechol- and SU-free control. (**B**) Representative images of DPPH assay wells. Statistical significance was analyzed using one-way ANOVA with Tukey’s post hoc test (* *p* < 0.05, *** *p* < 0.001; NS, not significant).

Beyond antimicrobial protection, the antioxidant activity of the hybrid hydrogels was evaluated by 2,2–diphenyl-1-picrylhydrazyl (DPPH) radical scavenging assay (Figure 12). As expected, the catechol-free MeHA exhibited minimal radical scavenging activity, whereas HA-Cat hydrogels showed significantly enhanced antioxidative capacity, consistent with the intrinsic redox–active catechol chemistry that could scavenge reactive radical species [19,41,42]. Notably, the incorporation of SU or SU@SAG did not significantly alter antioxidant performance relative to HA-Cat alone, suggesting that radical scavenging activity is predominantly governed by the catechol-functionalized polymer network, rather than the inorganic component or osteoinductive cargo. Representative visual DPPH assay imagery further supported these quantitative findings through clear discoloration in catechol-containing hydrogel groups.

Collectively, the hybrid architecture integrates SU-mediated antimicrobial defense with catechol-driven antioxidative protection, establishing a multifunctional protective microenvironment suited to tissue regeneration. These coordinated biological functions are particularly relevant for biomaterial-assisted bone regenerative applications, where microbial contamination and excessive oxidative stress can compromise tissue integration and destabilize the biomaterial interface [43,44]. Together, the organic and inorganic components cooperatively provide structural support, antimicrobial protection, redox regulation, and osteogenic functionality, highlighting the multifunctional potential of this injectable hybrid platform for bone regenerative biomaterial applications.

## 3. Conclusions

In summary, we developed a dynamically crosslinked organic–inorganic hybrid hydrogel by integrating catechol-functionalized HA with SU nanosheets as multifunctional inorganic junction domains. SU incorporation reorganized and reinforced the hydrogel network, resulting in reduced swelling, enhanced mechanical stability, delayed degradation, and preservation of injectability with self-recovery behavior. Beyond structural reinforcement, the hybrid hydrogel established a multifunctional microenvironment with antimicrobial and antioxidant properties. SU provided intrinsic osteoconductive activity, while incorporation of the osteoinductive small molecule SAG further enhanced osteogenic differentiation. Gene-expression analyses indicated coordinated modulation of Hedgehog- and Wnt/β-catenin-related markers, providing mechanistic insight into the enhanced osteogenic response. Collectively, this study presents a carbohydrate-based organic–inorganic network engineering strategy for structurally robust and biologically instructive injectable nanocomposite hydrogels with potential utility in bone regenerative biomaterial applications.

## 4. Materials and Methods

### 4.1. Materials

Sodium hyaluronate (weight-average molecular weight of 1,300 kDa; purity ≥98%) was sourced from Cosnet (Yecheon, Republic of Korea). Methanol (HPLC grade), dopamine hydrochloride, and SIGMAFAST™ BCIP^®^/NBT were purchased from Sigma–Aldrich (St. Louis, MO, USA). Sumecton–SA (Sumecton clay, SU) was kindly supplied by Kunimine Industries Co., Ltd. (Tokyo, Japan). Cell Counting Kit–8 (CCK-8) was obtained from APExBIO (Houston, TX, USA), while Smoothened agonist (SAG) was purchased from Cayman Chemical (Ann Arbor, MI, USA). Alizarin Red S solution was acquired from Samchun Chemicals (Seoul, Republic of Korea). Tween 20, Dulbecco’s modified Eagle’s medium (DMEM), fetal bovine serum (FBS), penicillin–streptomycin, and trypsin were procured from Welgene Inc. (Gyeongsan, Republic of Korea).

### 4.2. Synthesis of HA-Cat

HA-Cat was prepared by coupling dopamine to the carboxyl groups of HA through EDAC/NHS chemistry. HA was first dissolved in deionized water to obtain a 1% (*w*/*v*) solution (50 mL). EDAC hydrochloride (740 mg) and *N*-hydroxysuccinimide (NHS, 450 mg) were then introduced into the HA solution, and the mixture was stirred for 20 min to activate the carboxyl groups. Dopamine hydrochloride was subsequently added, and the reaction was continued for 3 h at room temperature while maintaining the pH at 5.5 with 1 N HCl. Following the reaction, low-molecular-weight reagents and reaction byproducts were removed by dialysis against deionized water for 48 h using a membrane with a molecular-weight cutoff of 3.5 kDa. The dialyzed product was freeze-dried for 48 h and stored at −50 °C until use.

Structural modification of HA after dopamine conjugation was examined by ^1^H NMR spectroscopy (JEOL 400 MHz, Tokyo, Japan) and FT-IR spectroscopy (FT/IR-4100 LE, JASCO, Tokyo, Japan). Catechol substitution was quantified from the ^1^H NMR spectrum using the integrated aromatic signals of the catechol group (3H) relative to the HA *N*-acetyl methyl signal at approximately 1.9 ppm (3H). This analysis yielded a catechol degree of substitution of approximately 15%. FT-IR spectra were additionally obtained to assess the chemical changes associated with dopamine conjugation.

### 4.3. Preparation and Characterization of SU@SAG

SU@SAG was produced through solvent-assisted incorporation of SAG into the SU nanosheets. SU (100 mg) was first dispersed in 8 mL of deionized water. Separately, 1 mg of SAG was dissolved in 1 mL of ethanol and subsequently mixed with 1 mL of deionized water. This SAG solution was introduced dropwise into the SU dispersion under vigorous stirring, and the mixture was continuously stirred overnight at room temperature. Solvent removal was then carried out by stirring at 30 °C under a fume hood until the liquid had completely evaporated. The dried material was redispersed in 10 mL of deionized water and sonicated to recover the SU@SAG film deposited on the vessel wall. The resulting suspension was centrifuged at 2500 rpm for 15 min, and the recovered solid was freeze-dried for 72 h and stored at −50 °C until further use.

Physicochemical characterization of SU and SU@SAG was performed by FT-IR spectroscopy (FT/IR-4100 LE, JASCO, Japan), thermogravimetric analysis (TGA; TGA 2, METTLER TOLEDO, Greifensee, Switzerland), and powder X-ray diffraction (XRD; MiniFlex600, Rigaku, Tokyo, Japan). For TGA, approximately 5 mg of each sample was placed in an alumina crucible and heated from 25 to 600 °C under nitrogen at 5 °C/min. XRD patterns were acquired using Cu-Kα radiation (λ = 1.5418 Å) at 40 kV and 30 mA with a scan rate of 10 °/min.

SAG release from SU@SAG and HA-Cat hydrogels containing 2% SU@SAG was evaluated under in vitro conditions. Free SAG was analyzed in parallel under the same conditions for comparison. Samples (*n* = 3) were immersed in 5 mL of 10 mM PBS (pH 7.4) containing 0.1% Tween 20 and maintained at 37 °C with agitation at 70 rpm. At predetermined time points, the release medium was collected and replaced with an equal volume of fresh buffer. Released SAG was quantified by HPLC-UV against a calibration curve prepared from known SAG concentrations. The analytical system consisted of a Waters 600 pump and controller, a Waters 717 autosampler, a Waters 486 UV detector, and a ZORBAX Eclipse XDB C18 column (Agilent, Santa Clara, CA, USA; 4.6 × 250 mm, 5 μm). Acetonitrile was used as the mobile phase at a flow rate of 1.0 mL/min. Cumulative release was expressed relative to the initial amount of SAG in each sample.

SAG loading in SU@SAG was quantified using the same HPLC-UV system. The incorporated SAG was recovered by sequential extraction with 0.1 M HCl followed by acetonitrile/HCl mixtures. The collected extracts were combined, and the SAG content was determined from the corresponding calibration curve. Loading efficiency was calculated from the cumulative amount of SAG recovered by extraction relative to the initial amount of SAG used for SU@SAG preparation:

(1) $$Loading\;efficiency\left(\%\right)=(\frac{Amount\;of\;SAG\;recovered\;from\;SU@SAG}{Initial\;amount\;of\;SAG\;used})\times 100,$$

### 4.4. Preparation and Physicochemical Characterization of the HA-Cat/SU Nanocomposite Hydrogels

A 4% (*w*/*v*) HA-Cat solution was prepared in 0.01 M PBS (pH 7.4), whereas SU and SU@SAG were separately dispersed in deionized water as 6% (*w*/*v*) stock suspensions. The polymer solution and the required amount of either SU or SU@SAG dispersion were combined to obtain the designated formulations at a final volume of 1 mL. Gelation was induced with 3 μL of sodium periodate (5 mg/mL), corresponding to an approximate NaIO_4_-to-catechol molar ratio of 0.006. After gelation, each construct was rinsed three times with PBS to reduce residual oxidant and other soluble unreacted species. The final HA-Cat content was maintained at 3.3% (*w*/*v*) for all formulations.

Hydration properties were determined after equilibrating the hydrogels in PBS for 24 h. Surface liquid was gently removed before recording the hydrated mass (*W_w_*). The same samples were then frozen at −50 °C, freeze-dried for 48 h, and weighed again to determine the dry mass (*W_d_*). Equilibrium water content was calculated as:

(2) $$Equilibrium\;water\;content\left(\%\right)=\left(\frac{W_{w}-W_{d}}{W_{w}}\right)\times 100,$$

where $W_{w}$ and $W_{d}$ refer to the weight of wet and dry hydrogels, respectively.

The swelling ratio was calculated from the swollen and dry masses according to:

(3) $$Swelling\;ratio=\left(\frac{W_{s}}{W_{d}}\right),$$

where $W_{s}$ and $W_{d}$ denote the swollen and dry weights, respectively.

Hydrolytic degradation was examined by maintaining the hydrogels in 0.01 M PBS (pH 7.4) at 37 °C for as long as 6 weeks, with fresh buffer supplied once per week. Samples collected at each designated time point were frozen at −50 °C, lyophilized for 48 h, and weighed. The remaining mass was expressed as:

(4) $$Residual\;weight\left(\%\right)=\left(\frac{W_{t}}{W_{0}}\right)\times 100,$$

where $W_{0}$ and $W_{t}$ denote the initial and remaining dry weights, respectively.

For microstructural and elemental examination, 250 μL hydrogel specimens with a diameter of 10 mm were frozen at −50 °C and lyophilized. The dried samples were mounted on aluminum stubs, sputter-coated, and examined by field-emission scanning electron microscopy coupled with energy-dispersive X-ray spectroscopy (FE-SEM/EDS; Axia ChemiSEM LoVac, Thermo Fisher Scientific, Waltham, MA, USA).

### 4.5. Rheological Characterization

Rheological behavior of the HA-Cat/SU nanocomposite hydrogels was examined at 25 °C using an MCR 302e rheometer (Anton Paar GmbH, Graz, Austria) equipped with a 25 mm parallel-plate geometry. Approximately 1 mL of each formulation was placed on the measuring plate and allowed to equilibrate for 10 min before testing. The linear viscoelastic region (LVR) was first identified by a strain sweep conducted at an angular frequency of 10 rad/s. Frequency-dependent behavior was then assessed within the LVR at 1% strain over an angular frequency range of 0.1–100 rad/s.

Recovery of the hydrogel network after large deformation was evaluated using alternating step-strain cycles between 700% and 1% strain while recording the storage modulus (*G*′). These measurements were carried out for HA-Cat, HA-Cat w/2% SU, and HA-Cat w/2% SU@SAG. The 2% SU formulation was chosen as the representative reinforced composition based on its mechanical performance. Three independently prepared hydrogel batches were used for all rheological measurements.

### 4.6. Cell Culture and Viability Assay

The murine D1 BMSC cell line (CRL-12424) was purchased from the American Type Culture Collection (ATCC, Manassas, VA, USA). Cells were cultured in DMEM containing 10% FBS and 1% penicillin–streptomycin and maintained at 37 °C under humidified conditions with 5% CO_2_.

Viability of BMSCs encapsulated in HA-Cat/SU nanocomposite hydrogels was examined on Days 1, 3, 7, 10, and 14 using the CCK-8 assay. At each time point, the cell-containing hydrogels were rinsed with PBS and exposed to culture medium supplemented with 10% (*v*/*v*) CCK-8 reagent for 3 h at 37 °C. The resulting absorbance was recorded at 450 nm using a Synergy 2 microplate reader (BioTek Instruments, Winooski, VT, USA). Values were expressed relative to the corresponding HA-Cat control at the same time point, which was assigned a value of 100%.

Cell survival within the hydrogel matrix was further examined after 7 d by Live/Dead staining. The encapsulated cells were labeled with a Live/Dead viability kit (Life Technologies Corporation, Carlsbad, CA, USA) and visualized using a Nikon ECLIPSE Ts2 fluorescence microscope.

### 4.7. ALP Assay

For ALP evaluation, cell-laden HA-Cat/SU nanocomposite hydrogels (100 μL per construct) were transferred to 24-well plates and maintained in 2 mL of growth medium for the first 24 h. Osteogenic differentiation was then initiated by replacing the medium with DMEM containing 10 mM β-glycerophosphate, 50 μg/mL L-ascorbic acid, and 100 nM dexamethasone. After 7 d, the constructs were fixed in 4% paraformaldehyde for 15 min and rinsed with PBS. ALP staining was carried out for 1 h at room temperature using Nitro Blue Tetrazolium/5-bromo-4-chloro-3-indolyl phosphate (NBT/BCIP) in ALP buffer, followed by imaging with an i-solution system.

For quantitative determination, the stained constructs were solubilized in dimethyl sulfoxide (DMSO) and homogenized, and the absorbance was recorded at 490 nm using a Synergy 2 microplate reader (BioTek). ALP values were expressed relative to the SU-free HA-Cat group. To correct for background signals from the clay-containing matrix, acellular SU-containing hydrogels were processed in parallel through the same staining and quantification steps, and their absorbance values were subtracted from those obtained from the corresponding cell-containing samples. The SAG concentration used in the SU@SAG-containing hydrogel group was 1 μM for all biological experiments.

### 4.8. Mineralization Assay

Matrix mineralization was assessed by Alizarin Red S staining after 14 d of osteogenic induction. Cell-laden HA-Cat/SU nanocomposite hydrogels were first fixed with 4% paraformaldehyde for 20 min and rinsed three times with deionized water. The samples were then exposed to 1% (*w*/*v*) Alizarin Red S solution (pH 4.2) for 60 min at room temperature. Unbound dye was removed by repeated washing with deionized water, and the stained constructs were documented using an i-solution imaging system and a Nikon ECLIPSE Ts2 microscope.

For quantitative measurement, retained Alizarin Red S was released from the hydrogels using 10% (*v*/*v*) acetic acid under gentle agitation. The resulting extracts were neutralized with ammonium hydroxide, and absorbance was recorded at 405 nm using a Synergy 2 microplate reader (BioTek). To account for nonspecific dye adsorption by the SU-containing matrix, acellular hydrogels were processed in parallel using the same staining and extraction protocol, and their absorbance values were subtracted from those of the corresponding cell-containing samples. Mineralization was expressed as fold change relative to the SU-free HA-Cat group.

### 4.9. Quantitative Real-Time PCR (qRT-PCR)

RNA was isolated from BMSCs embedded in HA-Cat/SU nanocomposite hydrogels using TRIzol reagent (Life Technologies Corporation, Carlsbad, CA, USA) and subsequently purified with an RNeasy Mini Kit (Qiagen, Valencia, CA, USA) following the manufacturers’ protocols. RNA quantity and purity were determined using a NanoDrop™ spectrophotometer (Thermo Fisher Scientific, Wilmington, DE, USA). For cDNA preparation, 0.5 μg of total RNA was reverse-transcribed with the SuperScript^®^ III First-Strand Synthesis System (Invitrogen, Carlsbad, CA, USA).

Quantitative PCR was carried out on a StepOnePlus Real-Time PCR System (Applied Biosystems, Foster City, CA, USA) using SYBR Green Master Mix. Each 20 μL reaction contained 1 μL of cDNA template. The amplification program comprised an initial 10 min denaturation at 95 °C, followed by 45 cycles of 95 °C for 15 s and 58 °C for 45 s. Relative transcript levels were determined by the 2^−ΔΔCt^ method using *GAPDH* as the internal reference and were expressed relative to the SU-free HA-Cat control group. Primer sequences used for the analysis are provided in Table 2.

### 4.10. Antimicrobial Activity Assay

Antimicrobial testing was conducted using *E. coli*, *S. aureus*, *A. niger*, and *P. notatum* as representative bacterial and fungal strains. Each microorganism was grown overnight in lysogeny broth (LB) at 37 °C, after which the cell density was adjusted according to the optical density at 600 nm (OD600). Hydrogel specimens were then exposed to the microbial suspensions for 24 h under the same incubation conditions.

After incubation, microbial growth was estimated from OD600 measurements obtained with a Synergy 2 multimode microplate reader (BioTek). The resulting values were expressed relative to the MeHA control. MeHA hydrogels, prepared according to a previously reported procedure [9], served as the catechol-free control in both the antimicrobial and antioxidant experiments.

### 4.11. Antioxidant Activity Assay

Antioxidant performance of the HA-Cat/SU nanocomposite hydrogels was determined using a DPPH radical scavenging assay. A fresh 0.25 mM DPPH solution was prepared in 95% ethanol. For each measurement, 200 μL of hydrogel sample was combined with 800 μL of the DPPH solution and kept in the dark for 30 min at room temperature with gentle agitation. The supernatant was then separated from the hydrogel and analyzed at 516 nm using a Synergy 2 multimode microplate reader (BioTek), thereby reducing interference from the hydrogel matrix.

Radical scavenging activity was determined as:

(5) $$Scavenging\;activity\left(\%\right)=\left(\frac{A_{o}-A_{s}}{A_{0}}\right)\times 100,$$

where *A*_0_ is the absorbance of the DPPH solution without hydrogel, and *A_s_* is the absorbance of the supernatant collected after incubation with the hydrogel sample.

### 4.12. Statistical Analysis

Data analysis was carried out with SigmaPlot 12.5 (Systat Software Inc., San Jose, CA, USA). Results are reported as mean ± standard deviation (SD), and *n* = 3 indicates three independently performed experiments unless stated otherwise. Depending on the comparison, either a two-tailed Student’s *t*-test or one-way ANOVA with Tukey’s post hoc multiple-comparison test was applied. A *p* value below 0.05 was considered statistically significant. Significance levels are denoted as NS, not significant; * *p* < 0.05; ** *p* < 0.01; and *** *p* < 0.001. Degradation data were analyzed by two-way ANOVA using hydrogel formulation and time as the two independent factors. For qPCR datasets, statistical testing was performed on ΔΔCt values, whereas the corresponding 2^−ΔΔCt^ fold-change values were used only for graphical display.

## Figures and Tables

**Table 1:** Elemental composition of HA-Cat/SU hybrid hydrogels determined by EDS. Atomic and weight percentages of major elements detected in HA-Cat hydrogels containing different SU formulations.

Element	HA-Cat		HA-Cat w/1% SU		HA-Cat w/2% SU		HA-Cat w/2% SU@SAG	
	Atomic %	Weight %	Atomic %	Weight %	Atomic %	Weight %	Atomic %	Weight %
C	30.5	24.6	32.7	25.8	28.3	21.5	30.4	23.5
O	69.1	74.4	62.3	65.5	63.1	63.8	62.1	63.8
Mg	0.0	0.0	2.5	3.9	2.1	3.2	3.3	5.2
Al	0.1	0.3	0.6	1.1	1.6	2.8	0.8	1.3
Si	0.3	0.6	2.0	3.7	4.9	8.7	3.4	6.2

**Table 2:** Sequences of primers for qPCR assay.

Primers	Forward	Reverse
*GAPDH*	AGGTCGGTGTGAACGGATTTG	TGTAGACCATGTAGTTGAGGTCA
*ALP*	GTTGCCAAGCTGGGAAGAACAC	CCCACCCCGCTATTCCAAAC
*RUNX2*	CGGTCTCCTTCCAGGATGGT	GCTTCCGTCAGCGTCAACA
*OCN*	GGGAGACAACAGGGAGGAAAC	CAGGCTTCCTGCCAGTACCT
*GSK-3β*	GCGTTCCCAAGAAGTGGCTTA	GGTCCAGCTTACGCATAATCTG
*β–catenin*	ATGGAGCCGGACAGAAAAGC	CTTGCCACTCAGGGAAGGA
*GLI1*	ACACATTACCAAGAAGCACCG	CAGCTGGTTTTCCCCTTTAAC
*PTCH*	AAACGGCTACCCTTTCCTGTTC	GACAATGATTCCAGCAGTCCAAG

## Data Availability

Data available on request from the authors.

## References

[1] Qu H., Fu H., Han Z., Sun Y. (2019). Biomaterials for bone tissue engineering scaffolds: A review. RSC Adv..

[2] Lewns F.K., Tsigkou O., Cox L.R., Wildman R.D., Grover L.M., Poologasundarampillai G. (2023). Hydrogels and bioprinting in bone tissue engineering: Creating artificial stem-cell niches for in vitro models. Adv. Mater..

[3] Amirazad H., Dadashpour M., Zarghami N. (2022). Application of decellularized bone matrix as a bioscaffold in bone tissue engineering. J. Biol. Eng..

[4] Lee C.-S., Singh R.K., Hwang H.S., Lee N.-H., Kurian A.G., Lee J.-H., Kim H.S., Lee M., Kim H.-W. (2023). Materials-based nanotherapeutics for injured and diseased bone. Prog. Mater. Sci..

[5] Hwang H.S., Lee C.-S. (2023). Recent progress in hyaluronic-acid-based hydrogels for bone tissue engineering. Gels.

[6] Hwang H.S., Lee C.-S. (2024). Nanoclay-composite hydrogels for bone tissue engineering. Gels.

[7] Yue S., He H., Li B., Hou T. (2020). Hydrogel as a biomaterial for bone tissue engineering: A review. Nanomaterials.

[8] Guo X., Li J., Wu Y., Xu L. (2024). Recent advancements in hydrogels as novel tissue engineering scaffolds for dental pulp regeneration. Int. J. Biol. Macromol..

[9] Song G.J., Oh S.-H., Lee J.H., Lee M., Hwang H.S., Koh J.-T., Lee C.-S. (2025). Photo-curable layered double hydroxide-hyaluronic acid-composite hydrogels with multifunctional properties for growth factor-free bone regeneration. Int. J. Biol. Macromol..

[10] Chen H., Xue H., Zeng H., Dai M., Tang C., Liu L. (2023). 3D printed scaffolds based on hyaluronic acid bioinks for tissue engineering: A review. Biomater. Res..

[11] Sekar M.P., Suresh S., Zennifer A., Sethuraman S., Sundaramurthi D. (2023). Hyaluronic acid as bioink and hydrogel scaffolds for tissue engineering applications. ACS Biomater. Sci. Eng..

[12] Serafin A., Culebras M., Collins M.N. (2023). Synthesis and evaluation of alginate, gelatin, and hyaluronic acid hybrid hydrogels for tissue engineering applications. Int. J. Biol. Macromol..

[13] Burdick J.A., Prestwich G.D. (2011). Hyaluronic acid hydrogels for biomedical applications. Adv. Mater..

[14] Noh I., Kim N., Tran H.N., Lee J., Lee C. (2019). 3D printable hyaluronic acid-based hydrogel for its potential application as a bioink in tissue engineering. Biomater. Res..

[15] Trombino S., Servidio C., Curcio F., Cassano R. (2019). Strategies for hyaluronic acid-based hydrogel design in drug delivery. Pharmaceutics.

[16] Zhou D., Li S., Pei M., Yang H., Gu S., Tao Y., Ye D., Zhou Y., Xu W., Xiao P. (2020). Dopamine-modified hyaluronic acid hydrogel adhesives with fast-forming and high tissue adhesion. ACS Appl. Mater. Interfaces.

[17] Chen J., Yang J., Wang L., Zhang X., Heng B.C., Wang D.-A., Ge Z. (2021). Modified hyaluronic acid hydrogels with chemical groups that facilitate adhesion to host tissues enhance cartilage regeneration. Bioact. Mater..

[18] Quan W.-Y., Hu Z., Liu H.-Z., Ouyang Q.-Q., Zhang D.-Y., Li S.-D., Li P.-W., Yang Z.-M. (2019). Mussel-inspired catechol-functionalized hydrogels and their medical applications. Molecules.

[19] Lee C.S., Hwang H.S., Kim S., Fan J., Aghaloo T., Lee M. (2020). Inspired by nature: Facile design of nanoclay–organic hydrogel bone sealant with multifunctional properties for robust bone regeneration. Adv. Funct. Mater..

[20] Zhang W., Wang R., Sun Z., Zhu X., Zhao Q., Zhang T., Cholewinski A., Yang F., Zhao B., Pinnaratip R. (2020). Catechol-functionalized hydrogels: Biomimetic design, adhesion mechanism, and biomedical applications. Chem. Soc. Rev..

[21] Yan G., Chen G., Peng Z., Shen Z., Tang X., Sun Y., Zeng X., Lin L. (2021). The Cross-Linking Mechanism and Applications of Catechol–Metal Polymer Materials. Adv. Mater. Interfaces.

[22] Saiz-Poseu J., Mancebo-Aracil J., Nador F., Busqué F., Ruiz-Molina D. (2019). The chemistry behind catechol-based adhesion. Angew. Chem. Int. Ed..

[23] Wang Q., Feng S., Long Y., Liu J., Zhu Q. (2026). Hydrogen Bonding-Driven Poly (vinyl alcohol)/Cellulose Nanofibril Hydrogels with AgNPs for Sensitive Motion Monitoring. ACS Appl. Polym. Mater..

[24] Tipa C., Cidade M.T., Borges J.P., Costa L.C., Silva J.C., Soares P.I. (2022). Clay-based nanocomposite hydrogels for biomedical applications: A review. Nanomaterials.

[25] Marin M.M., Ianchis R., Leu Alexa R., Gifu I.C., Kaya M.G.A., Savu D.I., Popescu R.C., Alexandrescu E., Ninciuleanu C.M., Preda S. (2021). Development of new collagen/clay composite biomaterials. Int. J. Mol. Sci..

[26] Kundu K., Afshar A., Katti D.R., Edirisinghe M., Katti K.S. (2021). Composite nanoclay-hydroxyapatite-polymer fiber scaffolds for bone tissue engineering manufactured using pressurized gyration. Compos. Sci. Technol..

[27] Stealey S.T., Gaharwar A.K., Zustiak S.P. (2023). Laponite-based nanocomposite hydrogels for drug delivery applications. Pharmaceuticals.

[28] Miao S., Zhou J., Lei X., Wang T., Cheng P., Pei G., Bi L. (2025). Optimal proportion of Laponite in hydrogel for promoting bone regeneration via MAPK-Erk pathway. Mater. Des..

[29] Cai F.F., Heid S., Boccaccini A.R. (2021). Potential of Laponite^®^ incorporated oxidized alginate–gelatin (ADA-GEL) composite hydrogels for extrusion-based 3D printing. J. Biomed. Mater. Res. Part B Appl. Biomater..

[30] Lukin I., Erezuma I., Garcia-Garcia P., Reyes R., Evora C., Kadumudi F.B., Dolatshahi-Pirouz A., Orive G. (2023). Sumecton reinforced gelatin-based scaffolds for cell-free bone regeneration. Int. J. Biol. Macromol..

[31] Lee H., Dellatore S.M., Miller W.M., Messersmith P.B. (2007). Mussel-inspired surface chemistry for multifunctional coatings. Science.

[32] Lee C.-S., Kim S., Fan J., Hwang H.S., Aghaloo T., Lee M. (2020). Smoothened agonist sterosome immobilized hybrid scaffold for bone regeneration. Sci. Adv..

[33] Gorojankina T., Hoch L., Faure H., Roudaut H., Traiffort E., Schoenfelder A., Girard N., Mann A., Manetti F., Solinas A. (2013). Discovery, molecular and pharmacological characterization of GSA-10, a novel small-molecule positive modulator of Smoothened. Mol. Pharmacol..

[34] Sui H., Wu Z., Xiong Z., Zhang H., Heng B.C., Zhou J. (2025). Spatiotemporal application of small molecules in fracture healing. Biomater. Transl..

[35] Bertsch P., Diba M., Mooney D.J., Leeuwenburgh S.C. (2022). Self-healing injectable hydrogels for tissue regeneration. Chem. Rev..

[36] Gaharwar A.K., Peppas N.A., Khademhosseini A. (2014). Nanocomposite hydrogels for biomedical applications. Biotechnol. Bioeng..

[37] Chen C., Li Z., Xu C., Kang M., Lee C.S., Aghaloo T., Lee M. (2024). Self-assembled nanocomposite hydrogels as carriers for demineralized bone matrix particles and enhanced bone repair. Adv. Healthc. Mater..

[38] Haraguchi K., Takehisa T. (2002). Nanocomposite hydrogels: A unique organic–inorganic network structure with extraordinary mechanical, optical, and swelling/de-swelling properties. Adv. Mater..

[39] Miller M.Q., McColl L.F., Arul M.R., Nip J., Madhu V., Beck G., Mathur K., Sahadeo V., Kerrigan J.R., Park S.S. (2019). Assessment of hedgehog signaling pathway activation for craniofacial bone regeneration in a critical-sized rat mandibular defect. JAMA Facial Plast. Surg..

[40] Lee C.-S., Fan J., Hwang H.S., Kim S., Chen C., Kang M., Aghaloo T., James A.W., Lee M. (2023). Bone-targeting exosome mimetics engineered by bioorthogonal surface functionalization for bone tissue engineering. Nano Lett..

[41] Zhang H., Wang J., Hu H., Ma L. (2025). A highly transparent dopamine-copolymerized hydrogel with enhanced ROS-scavenging and tissue-adhesive properties for chronic diabetic wounds. Acta Biomater..

[42] Lu H., Liu J., Yu M., Li P., Huang R., Wu W., Hu Z., Xiao Y., Jiang F., Xing X. (2022). Photothermal-enhanced antibacterial and antioxidant hydrogel dressings based on catechol-modified chitosan-derived carbonized polymer dots for effective treatment of wound infections. Biomater. Sci..

[43] Marsell R., Einhorn T.A. (2011). The biology of fracture healing. Injury.

[44] Metsemakers W.-J., Morgenstern M., McNally M., Moriarty T., McFadyen I., Scarborough M., Athanasou N., Ochsner P., Kuehl R., Raschke M. (2018). Fracture-related infection: A consensus on definition from an international expert group. Injury.

